# Single-cell genetic models to evaluate orphan gene function: The case of QQS regulating carbon and nitrogen allocation

**DOI:** 10.3389/fpls.2023.1126139

**Published:** 2023-03-27

**Authors:** Lei Wang, Andrew J. Tonsager, Wenguang Zheng, Yingjun Wang, Dan Stessman, Wei Fang, Kenna E. Stenback, Alexis Campbell, Rezwan Tanvir, Jinjiang Zhang, Samuel Cothron, Dongli Wan, Yan Meng, Martin H. Spalding, Basil J. Nikolau, Ling Li

**Affiliations:** ^1^ Department of Biological Sciences, Mississippi State University, Mississippi State, MS, United States; ^2^ Roy J. Carver Department of Biochemistry, Biophysics, and Molecular Biology, Iowa State University, Ames, IA, United States; ^3^ Engineering Research Center for Biorenewable Chemicals, Iowa State University, Ames, IA, United States; ^4^ Center for Metabolic Biology, Iowa State University, Ames, IA, United States; ^5^ Department of Genetics, Development and Cell Biology, Iowa State University, Ames, IA, United States; ^6^ Mississippi School for Mathematics and Science, Columbus, MS, United States; ^7^ Institute of Grassland Research, Chinese Academy of Agricultural Sciences, Hohhot, China; ^8^ Department of Agriculture, Alcorn State University, Lorman, MS, United States

**Keywords:** single-cell systems, orphan gene *QQS*, NF-YC homologs, HAP2, HAP3, HAP5, carbon and nitrogen partitioning, *Chlamydomonas reinhardtii* and *Saccharomyces cerevisiae*

## Abstract

We demonstrate two synthetic single-cell systems that can be used to better understand how the acquisition of an orphan gene can affect complex phenotypes. The Arabidopsis orphan gene, *Qua-Quine Starch* (*QQS*) has been identified as a regulator of carbon (C) and nitrogen (N) partitioning across multiple plant species. *QQS* modulates this important biotechnological trait by replacing NF-YB (Nuclear Factor Y, subunit B) in its interaction with NF-YC. In this study, we expand on these prior findings by developing *Chlamydomonas reinhardtii* and *Saccharomyces cerevisiae* strains, to refactor the functional interactions between QQS and NF-Y subunits to affect modulations in C and N allocation. Expression of *QQS* in *C. reinhardtii* modulates C (*i.e.*, starch) and N (*i.e.*, protein) allocation by affecting interactions between NF-YC and NF-YB subunits. Studies in *S. cerevisiae* revealed similar functional interactions between QQS and the NF-YC homolog (HAP5), modulating C (*i.e.*, glycogen) and N (*i.e.*, protein) allocation. However, in *S. cerevisiae* both the NF-YA (HAP2) and NF-YB (HAP3) homologs appear to have redundant functions to enable QQS and HAP5 to affect C and N allocation. The genetically tractable systems that developed herein exhibit the plasticity to modulate highly complex phenotypes.

## Introduction

In the past two decades, the ever-increasing genomics data have revealed the occurrence of species-specific “orphan” genes that appear to provide a mechanism for generating evolutionarily disruptive (rather than adaptive) novel phenotypes (Carvunis et al., 2012; Arendsee et al., 2014; Singh and Wurtele, 2020; Li et al., 2021). Although genomes of all organisms, from bacteria to humans, harbor orphan genes, their biological roles in generating novel phenotypes are poorly understood. For example, orphan genes can typically comprise as much as 5-15% of the total genes in a genome (Arendsee et al., 2014; Li et al., 2015), yet many of them are uncharacterized (Arendsee et al., 2014). Orphan genes appear to provide novel mechanisms for regulating complex biological processes. For example, in plants, orphan genes can improve tolerance to stresses (Perochon et al., 2015; Bai et al., 2019; Li et al., 2019; Dong et al., 2022), or can be involved in soluble sugar metabolism (Jiang et al., 2020). *Qua-Quine Starch* (*QQS*), is one of the approximately 1,300 protein-coding orphan genes of Arabidopsis; thus, it does not share any significant sequence similarity to any gene in any other organism (Fischer and Eisenberg, 1999; Khalturin et al., 2009; Tautz and Domazet-Lošo, 2011; Silveira et al., 2013). Our prior work led to the discovery that *QQS* can modulate carbon (C) and nitrogen (N) allocation in plants (Li et al., 2009; Li and Wurtele, 2015; Li et al., 2015).

In biological systems, proteins account for the majority of the N that occurs in cells (~16%), whereas carbohydrates and lipids are C-rich and N-poor. Thus, C and N partitioning primarily reflects the balance between the biosynthesis and degradation of these three classes of biomolecules. Although many of the genes involved in the primary metabolism that contribute to maintaining the balance between proteins (N), carbohydrates and lipids (C) are phylogenetically conserved, the regulation of these systems is complex and diverse (Gutiérrez et al., 2007). Moreover, C and N partitioning is a highly complicated process that is critical for organisms to perform fundamental cellular activities. Hence, the system of C and N partitioning is controlled by multiple metabolic and regulatory genes (Wenefrida et al., 2013). Disruption of this regulation in plants that imbalance in C and N partitioning causes problems in growth and development, and ultimately affects the completion of the life cycle and the production of harvestable organs (Chen et al., 2015; Yuan et al., 2016). In animals, C and N imbalance increases susceptibility to diseases and causes such disorders as intellectual disability, stunting, wasting, and sometimes death; globally, these disorders affect hundreds of millions of children each year (Young and Pellett, 1994; Pimentel and Pimentel, 2003; Gomes et al., 2009; Forrester et al., 2012). A more complete understanding of how the regulation of metabolism contributes to the balance between proteins, carbohydrates and lipids in plants is of utility in order to design these systems by genetic modifications, and thereby improve the nutritional quality of food crops.

*QQS* was initially identified by the finding that its down-regulation in transgenic RNAi experiments altered starch content in Arabidopsis (Li et al., 2009). Analogous effects were subsequently demonstrated by the transgenic expression of *QQS* in crop species (*i.e.*, soybean, rice, corn, tobacco and potato) (Li et al., 2009; Li and Wurtele, 2015; Li et al., 2015; Tanvir et al., 2022a; Tanvir et al., 2022b). These studies also demonstrated that overexpression of *QQS* in Arabidopsis or its transgenic expression in soybean and tobacco affected multiple traits, stimulating defenses against insect pests (aphid, soybean cyst nematode and white fly) and pathogens (bacterium, virus, and fungus), without impairing plant growth and yield (Qi et al., 2019; Tanvir et al., 2022a). RNAi-mediated down-regulation of *QQS* expression in Arabidopsis had the opposite effect, increasing starch content and decreasing protein content (Li et al., 2009), and increasing susceptibility to pathogens (Qi et al., 2019). Additionally, RNA-Seq analysis of these RNAi lines identified alteration in the expression of defense and stress-responsive pathways (*i.e.*, the ascorbate glutathione pathway, salicylate glucosides biosynthesis pathway, cutin biosynthesis and nitrate reduction pathway (Qi et al., 2019)).

*In vivo* reciprocal yeast two-hybrid assays, confirmed by *in vivo* co-immunoprecipitation experiments in Arabidopsis and bimolecular fluorescence complementation (BiFC) in tobacco, together with pull-down assays, have established that *QQS* affects these phenotypes by physically interacting with one of the NF-YC subunit paralogs (*i.e.*, NF-YC4) of the NF-Y transcriptional regulator (Li et al., 2015; Qi et al., 2019). In yeast and mammals, the NF-Y complex regulates the transcription of many target genes, which affects a wide range of biological processes, such as cell proliferation and apoptosis, cancer and tumor development, stress responses, growth and cellular development (Hooft et al., 1990; Li et al., 1992; Nardini et al., 2013; Oldfield et al., 2019). In plants, the NF-Y complex regulates diverse functions during stress responses, growth, and development (Zhao et al., 2017).

NF-Y is a trimeric complex consisting of NF-YA, NF-YB and NF-YC subunits (Laloum et al., 2013; Nardini et al., 2013). In contrast to yeast and mammalian genomes, where single copy genes encode each of the NF-Y subunits, multiple genes encode each of these subunits in plant genomes (Laloum et al., 2013). For example, in Arabidopsis, the NF-YA, NF-YB and NF-YC subunits are encoded by 21, 27 and 21 gene models, respectively. Because of this genetic redundancy in plant genomes, there are a variety of combinations of NF-Y complexes that can be assembled (Zhao et al., 2017).

Two sequence motifs of the QQS protein sequence, situated between residues 5 and 11, and 41 and 49, mediate the binding of QQS to the NF-Y complex, specifically binding to the NF-YC subunit (Qi et al., 2019). Based on the high sequence homology between the QQS binding motifs and the region of NF-YB protein that binds with NF-YC subunits, it appears that the binding of QQS to NF-YC is mimicking the binding between the NF-YB and NF-YC subunits (Qi et al., 2019). These findings enabled the development of a mechanistic model for the action of this orphan gene, namely QQS affects phenotypes by mimicking and replacing the NF-YB binding to the NF-YC subunit, enabling the formation of novel NF-Y complexes (Qi et al., 2019). This mechanistic model was developed based on experimental data generated with one of the 21 paralogs of the NF-YC subunit encoded by the Arabidopsis genome (*i.e.*, NF-YC4) (Qi et al., 2019). Therefore, because of genome complexity, it is unclear how the other 20 NF-YC paralogs could participate in the formation of this novel complex, and it is unclear how different NF-YA and NF-YB paralogs participate in these interactions.

Because of the complexity of multicellular organisms, combined with the genetic redundancy that is found in higher plants (*i.e.*, multiple genes coding for the NF-Y subunits), in this study, we redeployed this system in two genetically simpler unicellular eukaryotic organisms, *S. cerevisiae* and *C. reinhardtii*. These model organisms offer multiple advantages to dissect the role of *QQS* in regulating C and N allocation, and thereby provide a biological model vehicle to decipher the mechanisms by which, orphan genes hijack regulatory function, providing a pathway for generating disruptive novel phenotypes.

## Materials and methods

### Sequence alignment and phylogenetic analysis

Amino acid sequences of NF-YC homologs were analyzed using the CLUSTALW software from the Kyoto Encyclopedia of Genes and Genomes (KEGG) (https://www.genome.jp/tools-bin/clustalw), with standard parameters (*i.e.*, Gap Open Penalty: 10, and Gap Extension Penalty: 0.05). The neighbor-joining tree was constructed using MEGA version X based on the JTT+G+F model (Kumar et al., 2018). All positions containing gaps and missing data were eliminated for analysis. Bootstrap values were calculated from 1000 trees. The percentage of replicate trees in which the associated taxa clustered together in the bootstrap test are shown next to the branches.

### *Chlamydomonas reinhardtii* growth conditions

A plus mating type of the *C. reinhardtii* wild-type (WT) 21gr strain were used as controls. The 21gr (*mt*+) WT strains and the *QQS* transgenic lines were grown photoautotrophically in 200-mL photobioreactors containing a minimal growth medium consisting of 10 mM urea, 1.22 mM K_2_HPO_4_, 0.76 mM KH_2_PO_4_, 0.405 mM MgSO_4_·7H_2_O, 0.34 mM CaCl_2_·2H_2_O, 1 mL/L of Hutner′s trace elements, and buffered with 20 mM MOPS titrated with Tris to pH 7.3 (Xie et al., 2014). Cultures were aerated with a mixture of 5% CO_2_ in air and continuously illuminated with 6500 K fluorescent lamps at an intensity of 300 μmol photons·m^−2^·s^−1^ PAR under the continuous illumination. Photobioreactor cultures were initiated at a cell density of 1 x 10^5^ cells/mL, maintained at 25°C, and grown to stationary phase (6 days). Growth was monitored by measuring both optical density at 750 nm and by counting cell density using a Z1 Coulter Particle Counter (Beckman Coulter Inc. USA). Samples were collected at 2-, 4-, and 6-day intervals for dry weight, protein, and starch analysis.

### Construction of expression plasmids for *Chlamydomonas reinhardtii*

For *QQS* expression in *C. reinhardtii*, the *AtQQS* ORF (Open Reading Frame) was PCR-amplified from QQSpB2GW7 (Li et al., 2015) with specific primers (**Supplementary Table S1**) that introduced an *Nde*I site overhanging the translational ATG start codon at the 5′ end, and an *Eco*RI site after the stop codon at the 3′ end. The amplified DNA was digested by *Eco*RI and *Nde*I and ligated into the corresponding sites of the pGenD plasmid (Fischer and Rochaix, 2001), which placed the *QQS* ORF between the constitutive *PsaD* promoter and *PsaD* terminator. A *Hin*dIII/*Kpn*I DNA fragment containing the AphVIII selection marker from pSI103-delta (Sizova et al., 2001) was inserted downstream of the *PsaD* terminator to complete the final R15-QQS plasmid. This plasmid was linearized and used to transform *C. reinhardtii, strain 21gr (CC-1690, Chlamydomonas Resource Center, USA)*.

### Transformation and gene expression analysis in *Chlamydomonas reinhardtii*

WT 21gr cells grown mixotrophically in liquid TAP medium were harvested at the early log phase (0.5-1 X 10^6^ cells/mL) and directly used for electroporation without the autolysin-mediated removal of the cell wall. Electroporation was performed as described (Shimogawara et al., 1998) with a Gene Pulser Xcell electroporator (BioRad, USA; the condition was set as 650 V, 25 µF, 0 resistance with an exponential pulse). After electroporation, cells were resuspended in 15-mL TAP medium, and recovered for 24 h with gentle agitation (150 rpm) before plating on TAP plates supplemented with 15 µg/mL paromomycin for selection. Colonies grown under selective conditions were transferred to fresh TAP plates for further gene expression analysis. First, the presence of *QQS* sequence integrated into the genome in the putative transformants was detected by colony PCR with a specific primer complementary to the *PsaD* promoter sequence and a primer complementary to the *QQS* coding sequence (**Supplementary Table S1**). To detect *QQS* mRNA in the transformants bearing *QQS* transgene, each transformed cell line was grown in a liquid TAP medium, and RNA was isolated as previously described (Li et al., 2015), using TRIzol reagent (Invitrogen, USA) and purified with RNeasy-mini-Qiagen kit (Qiagen, USA), and treated with DNase I. Complementary DNA was synthesized using M-MLV Reverse Transcriptase (New England BioLabs, USA). Subsequently, RT-PCR was performed for *QQS* transcript analysis. *CBLP* (encoding guanine nucleotide-binding protein beta subunit-like protein) gene was used as the internal control (Fang et al., 2012). Assays were conducted on at least three biological replicates. The primers used in these analyses are listed in **Supplementary Table S1**.

### Extraction and quantification of starch and protein in *Chlamydomonas reinhardtii*

Dry weight was determined after centrifuging 10 mL of cells at 4800 x *g* for 10 min, decanting the supernatant, washing cells once with distilled water, and transferring cells to a pre-weighed aluminum tray to be placed in a 75°C oven for 48 h. Starch content was determined as previously described in Fan et al. (2012). Protein content was measured by mixing 0.1 M NaOH with culture at a ratio of 3:1, heating at 95°C for 30 min. After centrifugation at 22,000g for 1 min, the supernatant was recovered and protein concentration was determined by using the Pierce BCA Protein Assay Kit (Fisher Scientific, USA) (Bigelow et al., 2014).

### Yeast growth conditions

WT yeast strains were cultured in YPD (1% yeast extract, 2% peptone, 2% dextrose from Sigma-Aldrich, USA) media as described (Treco and Lundblad, 2001). Strains carrying kanMX disrupted mutant alleles were selected using YPD media containing 200 mg/L G418. Synthetic defined (SD) media was used to culture and select yeast strains containing plasmids that complimented auxotrophic mutant alleles (*ura3* or *his3*). The media was composed of 0.2% drop-out mix (minus uracil and/or histidine), 0.17% yeast N base (minus amino acids) and 2% dextrose (Sigma-Aldrich, USA). All strains were cultured at 30°C in liquid media or on 2% agar plates.

### Construction of hap mutant strains in yeast

*S. cerevisiae* haploid strain BY4741, mating type a (*MAT*a), and haploid strain BY4742 mating type α (*MAT*α), carrying *hap2, hap3, hap4*, or *hap5* mutant alleles were obtained from the American Tissue Culture Collection (www.atcc.org), and these strains were the products of Saccharomyces Genome Deletion Project (www.sequence.stanford.edu/group/yeast_deletion_project/deletions3.html). Mutant strains carrying multiple Knock-out (KO) alleles of the HAP complex were generated by mating and sporulating single *hap* KO strains, as previously described (Giaever and Nislow, 2014). *MAT*a and *MAT*α strains were grown on YPD-agar plates for 24 to 48 h at 30°C. A single colony of each mating type was mated on a YPD plate and grown at 30°C for 4 h. A patch of cells was then transferred from the mating plate to an SD (Lys- Met-) plate and grown for 24 h at 30°C. The cells were transferred to GNA pre-sporulation plates (5% D-glucose, 3% Difco nutrient broth, 1% Difco yeast extract) and grown at 30°C for 24 h. The cells were transferred to a new GNA plate and grown for 24 h at 30°C. A patch of cells was then transferred to 1-mL sporulation media (1% potassium acetate, 0.006% zinc acetate, 0.2% drop-out mix [met- lys-]) in sterile tubes. Sporulation tubes were placed in a room temperature shaker for 5 days, then transferred to a 30°C shaker for 3 days. Twenty-μL of 1 mg/mL Zymolyase was added to 100 μL suspension of sporulated cells in a 1.5-mL Eppendorf tube. Tubes were incubated at 37°C for 5-10 min and were then placed on ice. Twenty-μL suspension of the treated cells was pipetted onto the top half of a YPD plate. Tetrads were visualized using a dissection microscope. A total of 8–10 sets of tetrad spores were separated from each other and placed on the empty half of the YPD plate. The plate was placed in a 30°C incubator for 24-48 h, and the resulting yeast colonies were saved for testing of mating type.

The mating type of each colony was tested by the use of a “halo effect” induced by the use of the *Δbar1* strain (Manney, 1983). Patches of each colony were transferred to a YPD plate previously spread with a lawn of *Δbar1* colonies. Plates were incubated in a 30°C incubator for 24 h. Strains of *MAT*a type displayed a halo effect; *Δbar1* yeast did not grow on top of the patches of *MAT*a yeast, resulting in a circle of no growth around the patches. *MAT*α strains can be determined by observing lawn growth of *Δbar1* on top of the *MAT*α patches of yeast.

The *hap2*, *hap3, hap4*, and *hap5* KO strains were obtained as *MAT*a type, and thus were mated with BY4742 to generate single KO strains with the *MAT*α mating type. Single KOs were mated with each other and sporulated to generate tetrads containing double KOs of the HAP complex. Double KOs were then mated with each other and sporulated to generate tetrads containing the triple and quadruple KOs of the HAP complex. All spores were tested using PCR and gel electrophoresis analysis of the amplified DNA product to confirm the KOs of *hap2*, *hap3*, *hap4* and/or *hap5*.

### Expression of *QQS* and *HAP5* in yeast strains

The Arabidopsis *QQS* or yeast *HAP5* ORFs were cloned from entry vectors (pDONR221) into pAG destination vectors (Alberti et al., 2007) using the LR reaction as described by the product manual of Gateway^™^ LR Clonase^™^ II Enzyme mix (Fisher Scientific, USA). The destination vectors were isolated from the bacterial cells using the PureLink^™^ Quick Plasmid Miniprep Kit (Fisher Scientific, USA). The pAG destination vectors were pAG426 (high copy vector, carrying the URA3 auxotrophic marker), pAG416 (low copy vector, carrying the URA3 auxotrophic marker), pAG423 (high copy vector, carrying the HIS3 auxotrophic marker), and pAG423 (low copy vector, carrying the HIS3 auxotrophic marker). Expression from these episomal vectors was constitutive in the presence of glucose (GPD). The vectors also contain an ampicillin resistance gene for selection in *E. coli*.

The pAG plasmids containing *QQS* or *HAP5* ORFs were transformed into yeast cells using the lithium acetate/single-stranded carrier DNA/PEG rapid transformation method (Gietz and Stenback, 1998). The resulting cells were grown on SD media with either ura- and/or his- selection depending on the pAG plasmid that was used.

### Yeast genotyping

The presence of yeast mutant alleles and the presence of *QQS* and/or *HAP5* genes carried by pAG vectors were confirmed by PCR and gel electrophoresis analysis of the amplified DNA product. Primer sequences for these PCR-based genotyping experiments are listed in **Supplementary Table S1**.

All PCR reactions were performed using a mix of 12.5 μL GoTaq^®^ Hot Start Green Master Mix (Promega, USA), 0.25 μL of each primer at 10 μM concentration, 2 μL of DNA template and 10 μL ddH_2_O per reaction. The 25 μL reactions were performed in 8-well strips or 96-well plates. Aliquots of 5–15 μL of PCR product were analyzed by gel electrophoresis. The genotype for each strain was determined by comparing the observed fragment size to the expected size of the PCR products.

### Yeast growth phenotype

Overnight liquid cultures of each yeast strain were inoculated with a single colony into 5 mL of the rich YPD (Yeast Peptone Dextrose) media as specified previously (Rose et al., 1990; Guthrie and Gerald, 1991; Campbell et al., 2019). Once the cultures reached a sufficient optical density (OD_600_ > 0.5), they were diluted 1: 20 with fresh media, and 100 μL of the diluted culture was pipetted into a 96-well plate. The plates were then placed in a BioTek Eon^™^ spectrophotometer (BioTek Instruments, USA), with constant (double orbital) shaking until each strain reached stationary phase of growth (achieved by 36 h of growth). The OD_600_ for each strain was collected every 30 min. Doubling time was calculated to quantify the growth rate for each strain.

Growth rates were also determined in shake-flasks. Three independent colonies from three confirmed strains were inoculated into 5-mL starter cultures and grown overnight (OD_600_ > 1.5). The cultures were then diluted to OD_600_ = 0.1, and 50 mL of culture was grown in 250-mL Erlenmeyer flasks in a shaker at 30°C, and a 0.5-mL aliquot was removed every hour for the first 12 h and then every 3 h, to determine the OD_600_ for each culture. These data were used to determine the time required to reach the stationary phase. These data, in conjunction with the 96-well plate reader data, were used to identify the appropriate sample collection times for biochemical analyses.

### Growing yeast strains for biochemical analyses

**Figure 1:**
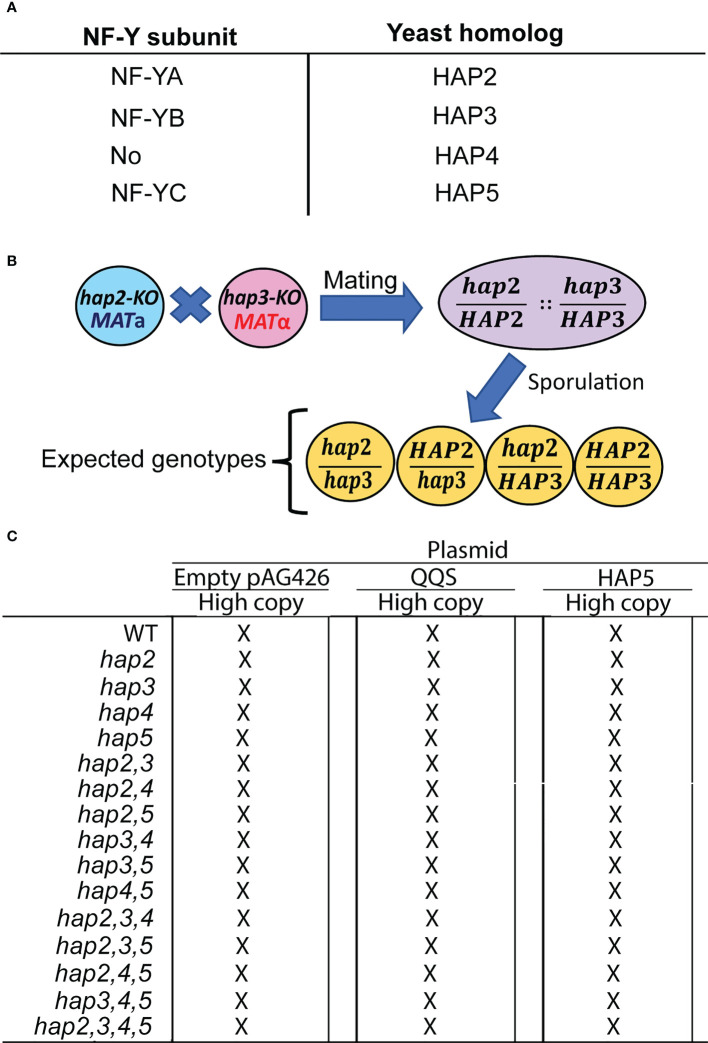
The strategy used to study the yeast *HAP* (*Heme-activated protein*) genes. **(A)** Identification of Arabidopsis NF-Y subunits and the homologous yeast HAP subunits. **(B)** The intermating genetic strategy for generating double mutant haploid yeast strains is exemplified by the generation of the *hap2hap3* double mutant strain; analogous strategies were used to generate all combinations of double, triple, and quadruple mutant strains. **(C)** The resulting 15 mutant strains and the WT strain were transformed with the indicated pAG426-based plasmid to generate *QQS-*transgenic strains (*QQS-E*) and *HAP5*-overexpressing strains (*HAP5-OE*).

Forty-eight unique yeast genotypes (**Figure 1C**) were subjected to biochemical analyses. Three biological replicates were analyzed per strain. Strains were grown in sets of 40, and the strain composition of each set was randomized. Culture samples from strains with a faster growth phenotype were collected after 15 h, while culture samples from strains with a slower growth phenotype were collected after 24 h of growth. This was done to ensure that all samples were collected at the early stationary phase. The culture samples from each flask were aliquoted into separate tubes depending on the type of metabolite extraction. The 50-mL culture was used for all of the metabolite extraction and quantification experiments.

### Extraction and quantification of fatty acids in yeast

At each chosen time-point in the early stationary phase of the yeast cultures, two 10-mL culture aliquots were removed and transferred to individual pre-weighed 15-mL Falcon tubes. Cells were pelleted by centrifugation at 4,000 x *g* for 5 min, the supernatant was removed, and pellets were flash frozen in liquid nitrogen, and stored at -80°C. Prior to extraction, samples were dried by lyophilization, and the dry weight of each cell pellet was determined.

Fatty acids were extracted using a sulfuric acid/methanol method (Dietrich et al., 2005; Perera et al., 2010; Quanbeck et al., 2012), and the order of these extractions was randomized. The dried pellets were transferred to screw-capped glass tubes, and each sample was spiked with 10 µg of nonadecanoic acid, which served as the internal standard for quantification purposes. Following the addition of glass beads and 1 mL of 5% sulfuric acid in methanol, the tubes were vortexed for 5 min and sonicated for 10 min. The screw-capped tubes were incubated at 80°C for an hour, and then allowed to cool to room temperature. The fatty acid methyl esters (FAMEs) were recovered by the addition of 1 mL of hexane:chloroform (4:1) and 1 mL of 0.9% sodium chloride solution. The tubes were vortexed for 5 min, and then centrifuged at 700 x *g* for 5 min to separate the aqueous and organic phases. The upper organic layer was collected and transferred to a GC vial, and the lower aqueous layer was re-extracted with two 1-mL aliquots of hexane:chloroform (4:1). The recovered organic phases were pooled and concentrated by evaporation, and the dried extracts were stored at -80°C. Prior to gas chromatographic (GC) analysis, the dried samples were resuspended in 300 µL of hexane:chloroform (4:1), and 1 µL of sample was injected into a GC equipped with a flame ionization detector (FID). A few samples were analyzed by GC-mass spectrometry (MS) in order to determine the chemical identity of the FAME peaks. Quantification was accomplished by comparing the peak areas of each FAME, with the peak area of internal standard FAME, and then summing the total amount of fatty acid detected for the C_12_-C_18_ FAMEs.

### Extraction and quantification of protein content in yeast

Two-mL aliquots of culture samples were collected in pre-weighed tubes at each chosen time-point, and cells were pelleted by centrifugation at 4000 x *g* for 5 min. After removing the supernatant, the tubes containing the cell pellets were flash frozen by submerging in liquid nitrogen, weighed, and stored at -80°C until extraction.

Protein extracts were prepared in sets of 80 samples. In order to reduce the variance introduced by extracting in separate sets, the 144 total samples were extracted in random order. Protein extraction was conducted using a DEST52 kit (Invitrogen Life Technologies, USA) following instructions provided by the manufacturer. The cell lysis buffer was modified by incorporating 2% (v/v) 2-mercaptoethanol and 0.1% (v/v) Triton-X100. The cell pellets were washed with 500 µL of lysis buffer and re-pelleted by centrifugation for 5 min at 1500 x *g* and 4°C. The supernatant was removed, cells were resuspended in 100 µL of lysis buffer, and an equal volume of acid-washed glass beads was added to the samples. Samples were vigorously vortexed for three 30-s periods, interrupted by a 30-s rest on ice and were centrifuged at 15,000 x *g* for 10 min. The supernatant was recovered into a new tube, and stored at -20°C.

Protein concentrations in each sample were measured using the Bradford colorimetric method (Bradford, 1976). These determinations were conducted in sets of 80 individual samples, and the order of samples was randomized. Twenty µl of each protein extract was pipetted into separate wells of a 96-well plate, and 200 µL of Bradford reagent was added to each well. Following a 15-min incubation at room temperature, the A_595_ of each well was determined using the BioTek Eon^™^ spectrophotometer (USA). Protein concentrations were calculated from a parallel generated standard curve using bovine serum albumin as the standard.

### Extraction and quantification of glycogen content in yeast

At each chosen time-point in the early stationary phase of the culture, two 5-mL aliquots of each culture were collected and transferred to a pre-weighed 15-mL Falcon tube. Following centrifugation at 4,000 x *g* for 5 min, the supernatant was removed, and the collected cell pellets were flash frozen in liquid nitrogen and stored at -80°C prior to extraction. Prior to extraction, the cell pellets were dried for 24 h by lyophilization, and the dry weight of the pellet was recorded.

To reduce the potential of introducing variance associated with the extraction protocol, glycogen extracts were prepared in sets of 40 samples, selected at random from the 144 total samples. Glycogen was extracted using an optimized hot alkali method (Gunja-Smith et al, 1977; Seo et al, 2002). Initially, cell pellets were washed twice with 1-mL aliquots of H_2_O to remove glucose that carried over from the culture medium. The washed pellets were suspended in 1 mL of 20% (w/v) potassium hydroxide solution and placed in a boiling water bath for 1 h. After cooling, the samples were adjusted to pH 6-7 using 5 N hydrochloric acid. Two volumes of 100% ethanol were added to each sample, and the precipitates were collected by centrifugation for 5 min at 4,000 x *g* at 4°C. The pellets were washed twice with 67% (v/v) ethanol, and the tubes were inverted over paper towels for at least 5 min to remove excess ethanol. The pellet was resuspended in 2 mL H_2_O by heating and vortexing each sample. Any undissolved material was pelleted by centrifugation for 5 min at 4,000 x *g* at 4°C, and the supernatant was collected to fresh 15-mL Falcon tube. Pellet was resuspended and washed with another aliquot of H_2_O, and following centrifugation, the supernatant was recovered and pooled. Polysaccharides in the pooled supernatants were precipitated by the addition of 1.5 volumes of 100% ethanol, and following incubation, on ice for 20 min, the precipitate was collected by centrifugation for 10 min at 4,000 x *g* at 4°C. The pellets were resuspended in 1 mL of H_2_O and flash-frozen in liquid nitrogen, and stored at -80°C. Once all 144 glycogen extracts were prepared, the order of samples for glycogen analysis was again randomized, and these analyses were conducted in sets of 80 samples.

Thawed glycogen extracts were adjusted to 10 mM sodium hydroxide and sonicated for 10 s. The glycogen in each sample (0.2-mL aliquots) was hydrolyzed in individual wells of a 96-well plate, with the addition of 30 µL of 100 mM sodium acetate, pH 5.0, containing 0.012 U of amyloglucosidase. The plates were incubated at 37°C for 2 h, and the concentration of the released glucose was quantified using the GAHK-20 Glucose Oxidase Assay Kit (Sigma-Aldrich, USA). At the start of these assays, a 66-µL aliquot was withdrawn from each assay and transferred to a new 96-well plate and the initial A_340_ of the solution was determined. Thirty-three µL of glucose oxidase reagent containing 1.5 mM NAD^+^, 1.0 mM ATP, 1.0 U/mL hexokinase, and 1.0 U/mL glucose-6-phosphate dehydrogenase was added to each well. The plate was placed in a spectrophotometer, and A_340_ of each assay was measured for a period of 1 h. A glucose standard curve was generated in parallel with each 96-well plate. Glucose content of each glycogen extract was quantified by relating the change in A_340_ to the change observed with the glucose standards.

### Statistical analysis

A minimum of three biological determinations from each independent transgenic line and each control were used for qualitative and quantitative analyses of composition. For compositional analyses, data are presented as mean ± SE (Standard Error). Statistical significance relative to the control was calculated with Student’s *t*-test (one tailed, not paired), or by analysis of variance (ANOVA).

## Results

The objective of this study was to develop a genetically calcitrant, single cell system that can be applied to investigate the genetic interactions between QQS and the NF-Y complex, which manifests altered phenotypes. Specifically, we were seeking to find single cell organisms whose genomes contain a smaller number of NF-Y subunit genes, which we could subsequently use to reconstitute the interactions between QQS and the NF-Y subunits.

### NF-Y homologs across species

**Figure 2:**
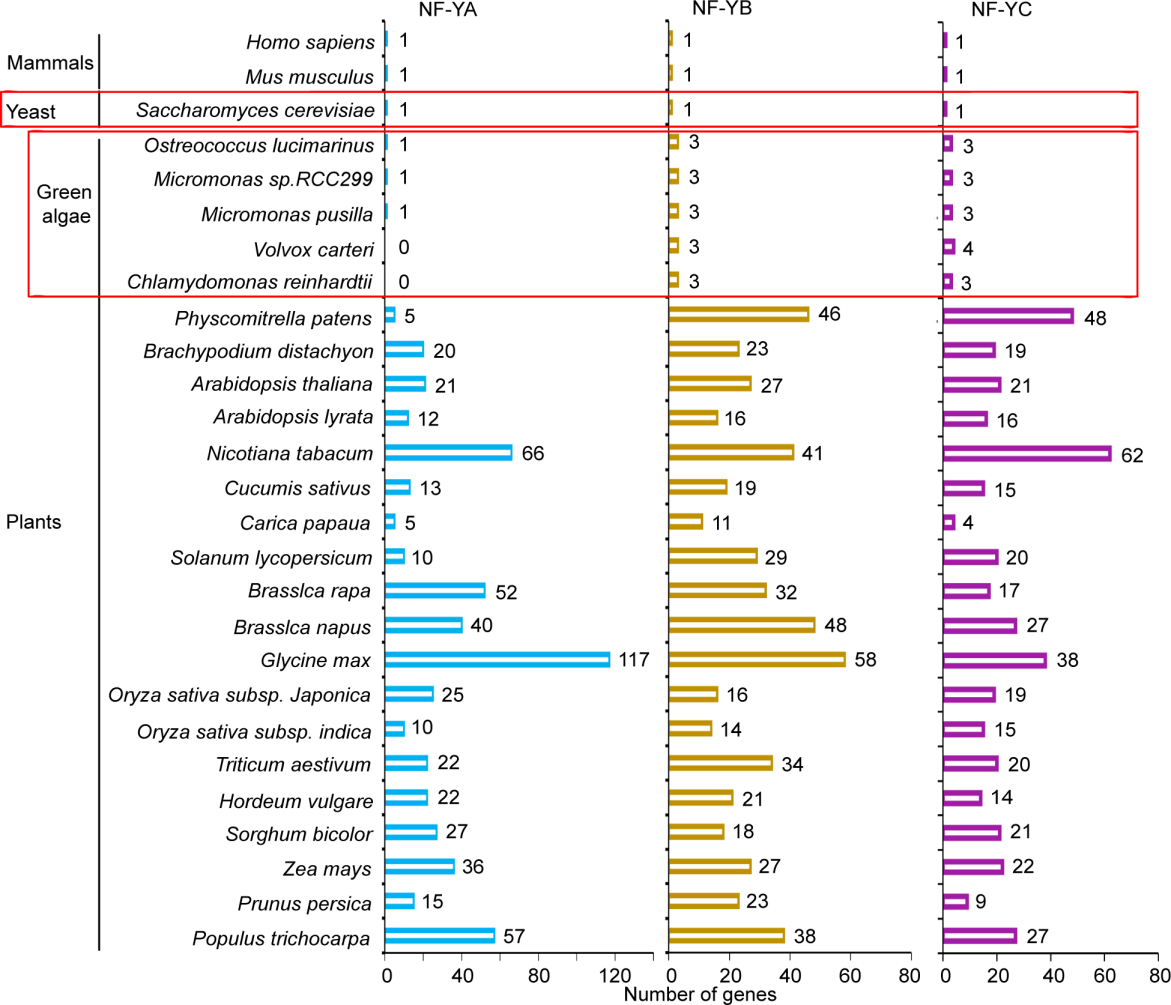
NF-Y subunit homologs in different species. Number of gene models of NF-YA, NF-YB, and NF-YC homologs in different species were identified from the plant transcription factor database, PlantRegMap/PlantTFDB v5.0 (http://planttfdb.gao-lab.org).

The plant transcription factor database, PlantRegMap/PlantTFDB v5.0 (Tian et al., 2020) was used to identify and analyze NF-YA, NF-YB, and NF-YC homologs in plants. As compared to mammals and yeast genomes, which encode single NF-Y subunit genes (Hooft et al., 1990; Nardini et al., 2013; Oldfield et al., 2019), higher plant genomes contain a large number of genes that encode these subunits (**Figure 2**). This is typified by the situation in the Arabidopsis genome, which encodes 21 NF-YA, 27 NF-YB, and 21 NF-YC gene models. The genome organization of these genes is simplified, but also more intriguing in the green algae clades, such as the unicellular *C. reinhardtii* genome, which encodes three NF-YB homologs and three NF-YC homologs, but there is no identifiable NF-YA homolog (**Supplementary Tables S2** , **S3**). Interestingly, single NF-YA homologs are recognizable in other green algae, such as *Ostreococcus lucimarinus*, *Micromonas pusilla*, and *Micromonas* sp. RC299, while there are no sequence homologs of NF-YA in the multicellular green alga, *Volvox carteri*.

Initially, we explored the phylogenetic relationships among the NF-YC proteins that bind to QQS. In its natural host, Arabidopsis, QQS interacts with NF-YC4 (Li et al., 2015). We therefore constructed a neighbor-joining phylogenetic tree using sequences of QQS-binding domain (residues 73-162) of the NF-YC4 protein (Li et al., 2015) from seven species (*i.e.*, *S. cerevisiae*, *Homo sapiens*, *C. reinhardtii*, *A. thaliana*, *Oryza sativa*, *Glycine max*, and *Zea mays*) (**Supplementary Figure S1**, **Supplementary Table S4**). These proteins segregate into four major clades, and the clade that includes AtNF-YC4 also contains the single-copy NF-YC protein of *S. cerevisiae* and *H. sapiens* and one of the three NF-YC isoforms of *C. reinhardtii* (Cre12.g556400). The *S. cerevisiae* and *H. sapiens* NF-YC sequences are located in adjoining branches, reflecting the fact that these homologs share 53% amino acid identity. The three Arabidopsis isoforms, AtNF-YC9, AtNF-YC3, and AtNF-YC4, are also located close to each other, consistent with the finding that these three proteins are functionally redundant (Kumimoto et al., 2010). It is interesting to note that unlike most of the rice and maize NF-YC isoforms that are located in a distinct clade, away from the AtNF-YC4 isoform, the rice (LOC_Os3g14669) and maize NF-YC4 isoforms (GrmZm2g089812 in genome Version 2, now ZmCA5P11: Zm00001eb010770 in genome Version 5) that bind to QQS (Li et al., 2015) are located in a clade adjoining the AtNF-YC4 homolog. Based on this correlation, one may expect that the *C. reinhardtii* NF-YC4 homolog (Cre12.g556400), which is adjoining to AtNF-YC4 may bind to QQS (**Supplementary Figure S1**).

This hypothesis was supported by additional analyses of the amino acid sequences of these homologous proteins. The CLUSTALW multisequence alignment shown in **Supplementary Figure S2** illustrates that Cre12.g556400 (CrNF-YC4) has very high homology with the AtNF-YC4, specifically encompassing the QQS-binding domain; namely 98% similarity and 73% identity between residues 73-162 of AtNF-YC4. This hypothesis was therefore evaluated by the expression of *QQS* in *C. reinhardtii*.

### Heterologous transgenic expression of *QQS* in *Chlamydomonas reinhardtii* affects carbon and nitrogen allocation

**Figure 3:**
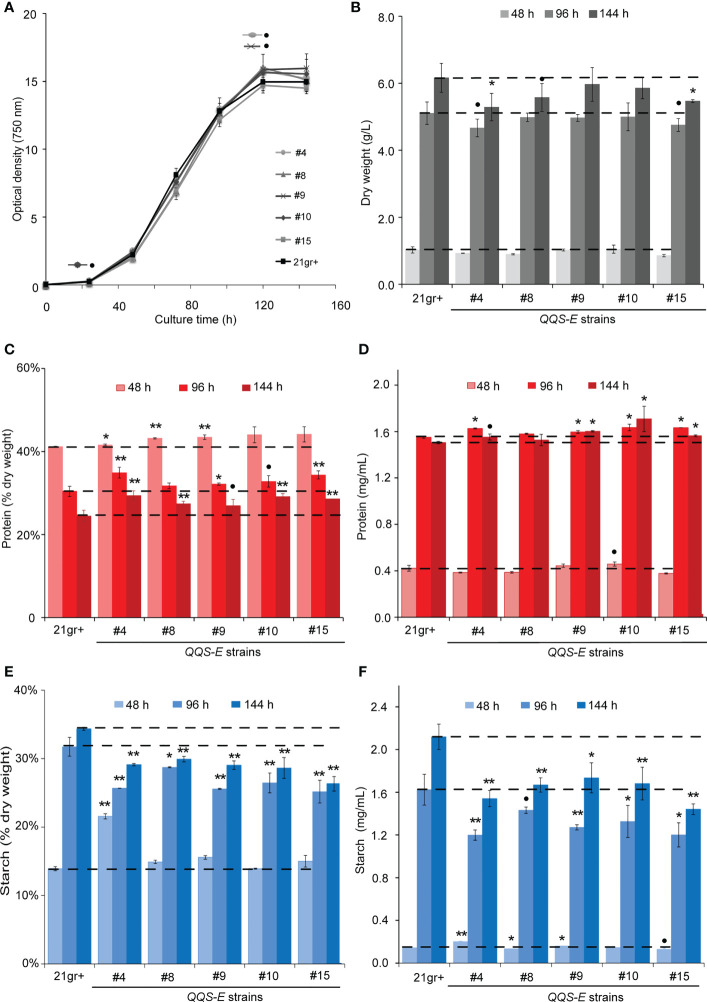
The effect of transgenic expression of *QQS* on the growth, starch and protein content in *Chlamydomonas reinhardtii*. Cultures were grown photoautotrophically to the stationary phase. Growth of the strains was determined by monitoring the optical density at 750 nm **(A)**, and biomass yield **(B)** of WT **(C)** *reinhardtii* (21gr^+^) and transgenic *QQS-E* strains at the indicated times after subculturing. **(C–F)** Protein and starch content of WT (21gr^+^) and transgenic *QQS-E* strains at the indicated times after subculturing. All data are mean ± SE (standard error), *n* = 3. Statistical significance relative to the WT strain was calculated with Student’s *t*-test and is indicated: **P < 0.01, *P < 0.05, •*P* < 0.1.

Five transgenic events were recovered from the transformation of *C.reinhardtii* with the *QQS* transgene. These recovered *QQS-E* strains were confirmed by PCR-based genotyping, and RT-PCR was used to confirm the expression of the *QQS* transcript (**Supplementary Figure S3**). The impact of *QQS* expression on the growth of *C. reinhardtii* was evaluated by monitoring the optical density (**Figure 3A**), cell density (**Supplementary Figure S4**) and dry weight biomass yield of cultures (**Figure 3B**) for all mutant strains as compared to WT. Collectively these analyses indicate that transgenic expression of *QQS* significantly affected the cell density as the cultures approached the stationary phase of growth (*i.e.*, only after 144 h), while it did not have a statistically significant impact on the growth of *C. reinhardtii* (at the *P* level of 0.05) as compared to the WT strain of 21gr^+^.

Parallel analyses of the protein and starch content showed that nearly all *QQS-E* strains had a statistically significant increase in protein levels as compared to the WT strain, the exception being *QQS-E* #8 (in mg/mL, interestingly this correlates with its *QQS* expression level in **Figures S3**) (**Figures 3C, D**). These changes in protein content depended on the stage of the cultures and ranged in increases of 2-8% at 48 h to 9-19% at 144 h (*P* < 0.01 for at least one time-point per *QQS-E* strain from the 48-h time-point onward, **Figure 3C**). Juxtaposed to this increase in protein content, starch content was decreased in the *QQS-E* strains, decreasing by up to 13-24% at 144 h (*P* < 0.01 for at least one time-point per *QQS-E* strain from the 96-h time-point onward; **Figures 3E, F**). Hence, these data establish that the transgenic expression of *QQS* affects C and N partitioning in *C. reinhardtii*, and this is accomplished even though this organism does not encode a recognizable NF-YA homolog.

### HAP5: The NF-YC homolog in Saccharomyces cerevisiae

To further evaluate the effect of QQS on C and N allocation, we used another genetically simpler, unicellular organism (*i.e.*, *S. cerevisiae*) as the host for similar transgenesis experiments. In *S. cerevisiae*, the NF-Y complex is known as the Heme-Activated Protein (HAP) complex, which is composed of the three structural subunits, HAP2, HAP3, and HAP5 (Olesen et al., 1987; Hahn and Guarente, 1988; Forsburg and Guarente, 1989; McNabb et al., 1995), homologs of NF-YA, NF-YB and NF-YC, respectively (**Figure 1A**). In addition, in yeast a fourth protein (HAP4) binds to the HAP2/HAP3/HAP5 DNA-binding complex, and stimulates transcription (McNabb and Pinto, 2005). In contrast, plant genomes do not encode a HAP4 homolog. Using this system therefore, we evaluated the ability of QQS to interact with HAP5, the NF-YC homolog. The multisequence alignment shown in **Supplementary Figure S2** identifies the homology between HAP5 and the Arabidopsis, *C.reinhardtii* and human NF-YC homologs, illustrating the conservation that is shared among these proteins in the QQS binding domain (Li et al., 2015); the yeast HAP5 protein shares 67% identity and 83% similarity with the QQS binding domain of AtNF-YC4.

### *QQS* expression and *HAP5* overexpression have a minor effect on the growth of yeast

We genetically evaluated if QQS interacts with the yeast HAP complex by expressing it or overexpressing *HAP5* subunit in different *S. cerevisiae* backgrounds that carried the knockout (KO) mutations in the HAP subunit genes, and compared the resulting strains to the control recipient strains (**Figure 1**). Mutant strains carrying KO mutant alleles in the four HAP subunit genes were obtained in both *MAT*a and *MAT*α mating types and these were all viable. Using an intermating strategy illustrated in **Figure 1B** for generating the *hap2,3* double mutant, we generated haploid strains that carried all possible combinations of the double mutant, triple mutant, and quadruple mutant strains (**Figure 1C**). The resulting collection of 15 mutant strains and the WT strain were subsequently recipients for three expression vectors. One of these was the pAG426 plasmid, which served as the control in all subsequent experiments. The other two vectors that were used in these experiments expressed either the *QQS* gene (*i.e.*, *QQS-E* strains) or the *HAP5* gene (*i.e.*, *HAP5-OE* strains), both being under the transcriptional regulation of the *GPD*-promoter that was carried by the pAG426 base-vector.

The resulting 48 strains (**Figure 1C**) were evaluated to ascertain the effect of these genetic manipulations on growth. Specifically, we monitored the rate of change in the optical density of cultures of each strain, from which doubling time was calculated. **Supplementary Figure S5A** plots the inverse of doubling times for the control and *hap2*, *hap3*, *hap4* and *hap5* single, double, triple and quadruple combination mutant strains; note all these strains carried the pAG426 empty vector plasmid specifically for the purpose of serving as the control strains for the *QQS-E* and *HAP5-OE* strains. The growth of all these mutant strains was indistinguishable from the WT (*i.e.*, *P* > 0.1).

We also determined the effect on cell growth when *QQS* was expressed (**Supplementary Figure S5B**) or *HAP5* overexpressed (**Supplementary Figure S5C**) in each of these mutant strains. The growth of the majority of these strains was unaffected by the expression or overexpression of either *QQS* or *HAP5*. The exceptions were the *hap2,4* double mutant strains, which showed a ~12% increase in growth in response to *QQS* expression (*P* = 0.03), whereas the *hap3* mutant strain showed a ~8% reduction in growth rate (*P* = 0.049). Although growth of additional mutant strains was statistically significantly affected by the overexpression of *HAP5* (namely the WT (*P* = 0.042), *hap2* (*P* = 0.041), *hap5* (*P* = 0.03), *hap2,4* (*P* = 0.02), *hap4,5* (*P* = 0.041), and *hap2,4,5* (*P* = 0.050)), in all cases, these effects were minor with only an 8 to 20% increase in growth rate.

### Reverse genetic dissection of the role of HAP subunit genes in the allocation of carbon and nitrogen

**Figure 4:**
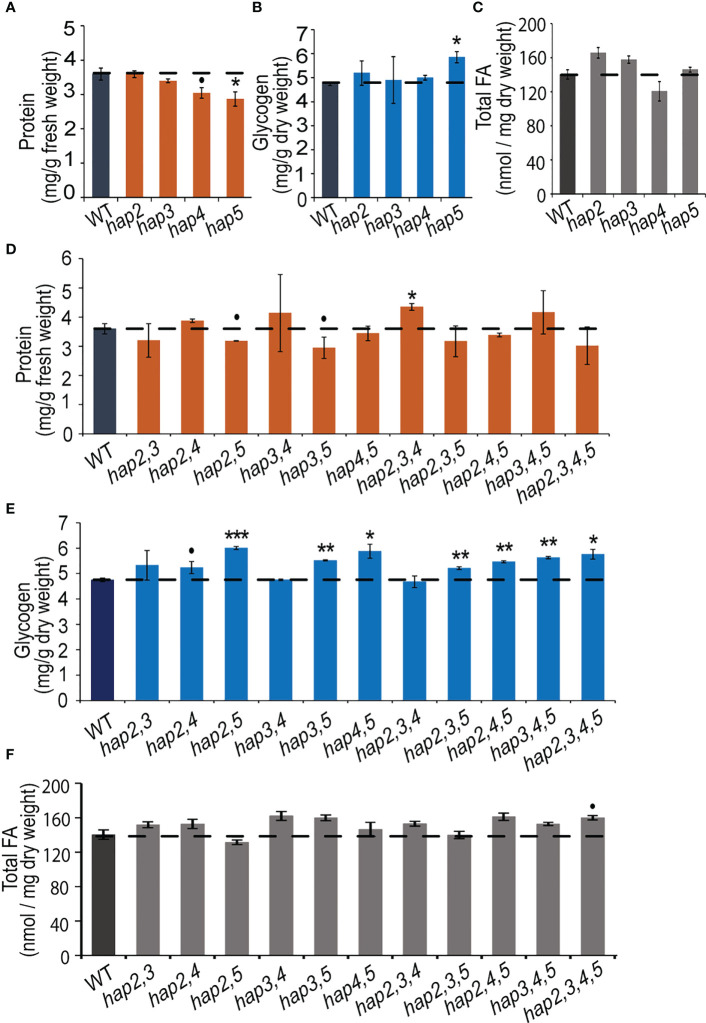
Protein, glycogen, and fatty acid content in yeast *hap* mutant strains. Protein **(A)**, glycogen **(B)**, and fatty acid **(C)** levels in yeast strains carrying *hap2*, *hap3*, *hap4*, or *hap5* mutations. Protein **(D)**, glycogen **(E)**, and fatty acid **(F)** levels in yeast strains carrying double, triple, and quadruple combinations of *hap2*, *hap3*, *hap4*, and *hap5* mutations. All data are mean ± SE, *n* = 3. Statistical significance relative to the WT strains was calculated with Student’s *t*-test and is indicated: ***P < 0.001, **P < 0.01, *P < 0.05, •*P* < 0.1.

In yeast, the major forms of C storage are glycogen and fatty acids, which together can account for approximately 10-20% of the wet weight of the cells (Quain and Tubb, 1983; Suutari et al., 1990; Cahill et al., 2000). To obtain an understanding of the role of the HAP complex in determining C and N allocation, we ascertained protein, glycogen and fatty acid content in yeast strains that carried *hap2*, *hap3*, *hap4* and/or *hap5* KO mutant alleles (**Figure 4**). The data shown in **Figures 4A, B** indicate that the *hap5* mutation has the most statistically consistent effect on protein and glycogen levels, increasing glycogen content by ~25% (*P* = 0.019) and decreasing protein content by a similar amount (~20%; *P* = 0.027). Parallel analyses of all potential double, triple and quadruple mutants reinforce the importance of the *HAP5* gene in regulating protein and glycogen levels (**Figures 4D, E**). Specifically, any of the double, triple and quadruple mutants that included the *hap5* mutation showed increased glycogen levels (10-20% increase) (**Figure 4E**), while some of these mutants also expressed decreased protein levels (*e.g.*, *hap2,5* and *hap3,5*) (**Figure 4D**). The parallel analysis of the fatty acid content in these KO mutants indicates that these HAP subunits do not appear to have statistically significant impacts in total fatty acid content (*P* > 0.05) (**Figure 4C**).

### The effect of expressing *QQS* or overexpressing *HAP5* in *Saccharomyces cerevisiae* on protein, glycogen, and fatty acid accumulation

**Figure 5:**
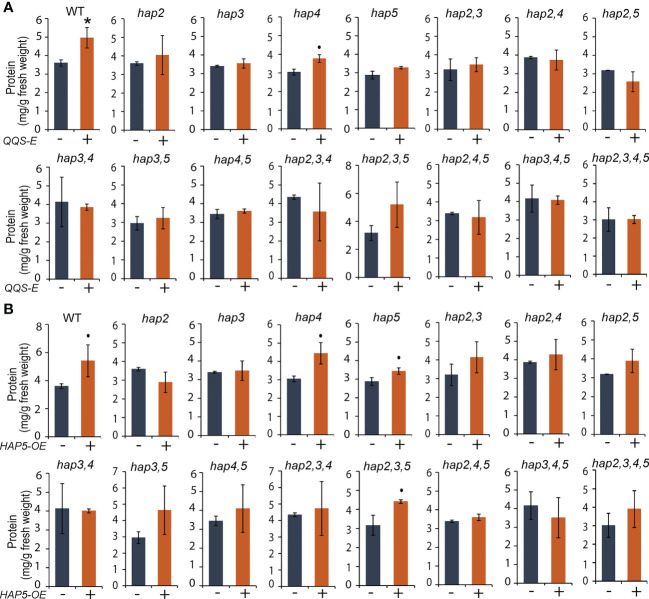
The effect of transgenic *QQS-E* and *HAP5-OE* on the protein content of yeast *hap* mutant strains. **(A)** *QQS-E*. **(B)** *HAP5-OE*. All data are mean ± SE, *n* = 3. Statistical significance relative to the strains without *QQS-E* or without *HAP5-OE* was calculated with Student’s *t*-test and is indicated: *P < 0.05, •*P* < 0.1.

**Figure 6:**
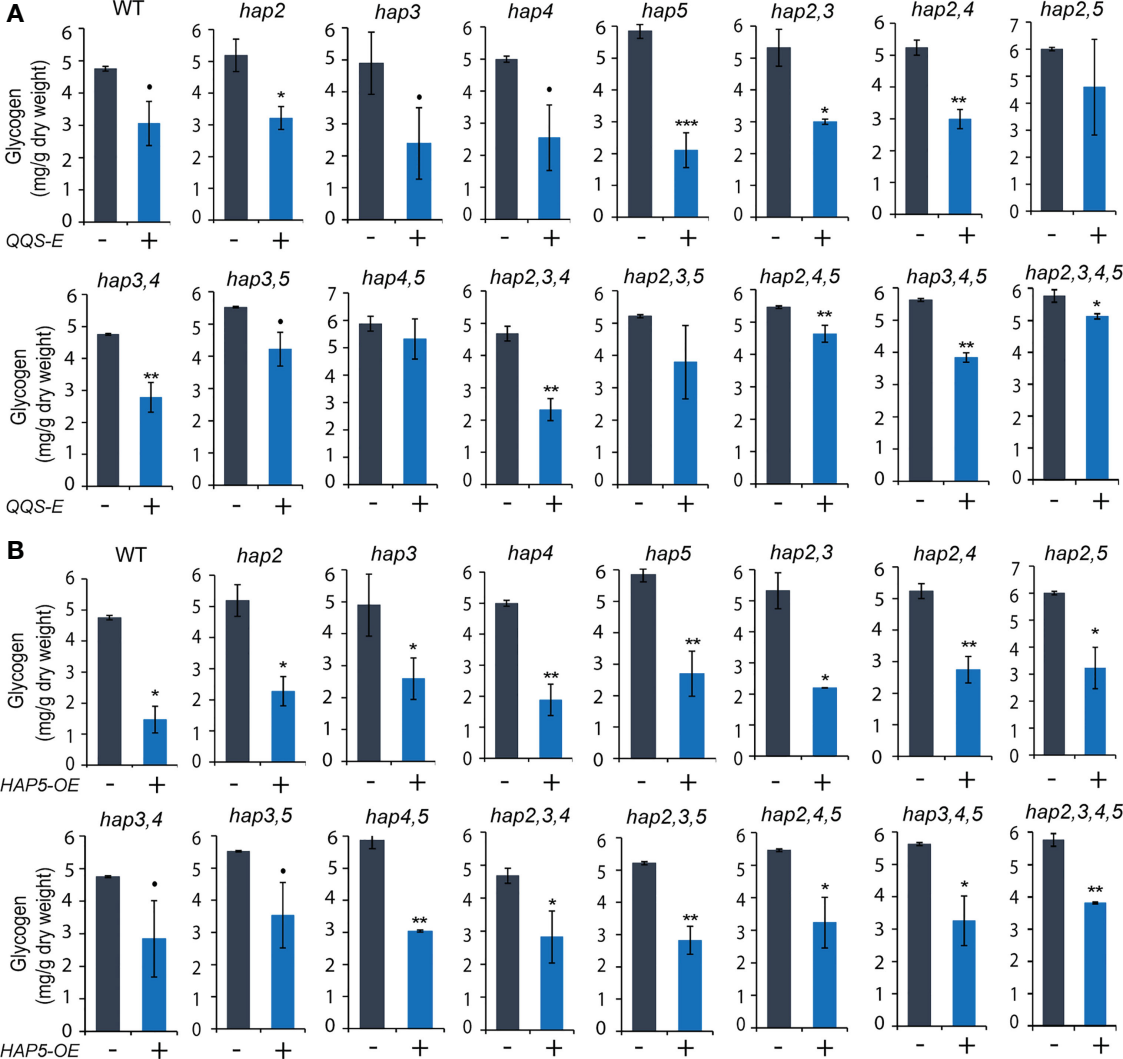
The effect of transgenic *QQS-E* and *HAP5-OE* on the glycogen content of yeast *hap* mutant strains. **(A)** *QQS-E*. **(B)** *HAP5-OE*. All data are mean ± SE, *n* = 3. Statistical significance relative to the strains without *QQS-E* or without *HAP5-OE* was calculated with Student’s *t*-test and is indicated: **P < 0.01, *P < 0.05, •*P* < 0.1.

The *QQS-E* and *HAP5-OE* strains developed in the WT background expressed a ~40% (*P* = 0.045) and ~50% (*P* = 0.095) increase in protein content, respectively (**Figures 5A, B**), and reduced glycogen content by similar amounts (**Figures 6A, B**). In contrast, these genetic manipulations had no significant impact on fatty acid content (**Supplementary Figure S6A, B**). These effects were further dissected by evaluating protein (**Figure 5**), glycogen (**Figure 6**), and fatty acid (**Supplementary Figure S6**) content in the *QQS-E* and *HAP5-OE* strains developed in genetic backgrounds that carried *hap2*, *hap3*, *hap4*, and *hap5* single, double, triple and quadruple KO mutant combinations. The effect of expressing *QQS* or *HAP5* on the fatty acid content of most of these mutant strains was statistically indistinguishable from the recipient strains (*P* > 0.1) (**Supplementary Figure S6**). The two exceptions were the ~15% increase in the fatty acid content of the *QQS-E* strain in the *hap3* mutant background (*P* = 0.062) and of the *HAP5-OE* strain in the *hap5* mutant background (*P* = 0.024).

However, the *QQS-E* and *HAP5-OE* strains in these mutant backgrounds showed distinct changes in protein content, indicating a role for HAP2 and HAP3, and possibly also for the HAP4 subunit. Specifically, protein content was increased by the expression of *QQS* or *HAP5* in the WT strain, and this modulation in protein content also occurs in the strains carrying the *hap4* KO mutant allele [*P* = 0.069 for *QQS-E* (**Figure 5A**), and *P* = 0.083 for the *HAP5-OE* strains (**Figure 5B**)]. However, because of somewhat larger variation in the data, we cannot exclude the possibility that a functional *HAP4* allele may be required to increase protein content by the expression of either *QQS* or *HAP5*. These increases in protein content did not occur in the *QQS-E* (*P* > 0.1) and *HAP5-OE* (*P* > 0.1) strains that carry either *hap2* or *hap3* KO mutant allele. Because prior studies had established that QQS mediates its effect by interacting with the NF-YC4 (HAP5) subunit (Li et al., 2015), it was expected that *QQS-E* could not increase protein content in the *hap5* KO mutant allele. However, the *QQS-E* and *HAP5-OE* in *hap2* or *hap3* KO mutants affected protein content, similar to in the *hap5* KO mutant. Therefore, these results suggest that in addition to the need for HAP5, the QQS-effect on protein content may also involve the HAP2 or HAP3 or potentially the HAP4 subunits.

The parallel analyses of glycogen content in these *QQS-E* (**Figure 6A**) and *HAP5-OE* (**Figure 6B**) strains indicate that the effect is more complex. Specifically, expressing *QQS* in any of the four *hap* single mutants decreased glycogen content by 38% in *hap2* (*P* = 0. 038), 51% in *hap3* (*P* = 0. 053), 49% in *hap4* (*P* = 0. 08), and 64% in *hap5* (*P* = 0. 001) (**Figure 6A**). However, when *QQS* was expressed in any combinations of the *hap2* or *hap3* KO alleles, where a WT *HAP5* allele was still active, glycogen levels were decreased; specifically, by 44% in *hap2,3* (*P* = 0.027), 43% in *hap2,4* (*P* = 0.002), 41% in *hap3,4* (*P* = 0.003) double mutants, and 50% in *hap2,3,4* (*P* = 0.003) triple mutant. Moreover, there was no statistically significant change in glycogen levels in *QQS-E* strains that combined the *hap5* KO mutant allele with any combination of *hap2* or *hap3* KO alleles; specifically, the *hap2,5* (*P* = 0.192), or *hap3,5* (*P* = 0.075) double mutants, or the *hap2,3,5* (*P* = 0.14) triple mutant. However, because of some larger variation in the data, we cannot exclude the possibility that a functional *HAP4* allele may be required to affect glycogen content. Collectively therefore, these results suggest that when HAP5 is absent, HAP2, HAP3 and probably HAP4 subunits may have redundant functions to enable QQS to affect glycogen levels. This is consistent with the finding that when *QQS* was expressed in the *hap2,3,4,5* quadruple mutant, the glycogen level was only reduced by ~10% (*P* = 0.046). The small change in glycogen level displayed by this quadruple mutant strain may be indicative of another unknown mechanism that enables *QQS* to affect the glycogen content in the absence of any HAP subunits.

Finally, overexpression of *HAP5* significantly decreased glycogen levels by between 30% and 70% in most strains (*P* < 0.05), except the *hap3,4* and *hap3,5* double mutants (*P* < 0.1), and these effects are consistent among all the different *hap* mutants that were evaluated, similar to the effect of overexpressing *HAP5* in WT yeast (**Figure 6B**). These effects of *HAP5* overexpression on glycogen content in yeast parallel our initial characterizations of *QQS* affecting starch content in plants, by activating the plant homolog of HAP5, namely AtNF-YC4 (Li et al., 2015).

## Discussion

Orphan genes are prevalent in a wide range of organisms, from bacteria to humans, and can account for a significant portion of the genome in any given species (Arendsee et al., 2014; Li et al., 2015). Yet the vast majority of these genes remain uncharacterized (Arendsee et al., 2014). It is becoming increasingly apparent that orphan genes, such as the Arabidopsis *QQS* gene (Carvunis et al., 2012; Arendsee et al., 2014; Singh and Wurtele, 2020; Li et al., 2021), or the *UP12_8740* gene of cowpea that confers drought resistance (Li et al., 2019), or the *TaFROG* gene of wheat that enhances resistance to disease by influencing cell signaling (Perochon et al., 2015), are an evolutionary force for generating novel phenotypes. The evolutionary emergence of orphan genes appears to provide an alternative, “step-change” in the evolution of a phenotype, complementary to the slower Darwinian adaptive-evolution of a phenotype (Carvunis et al., 2012; Arendsee et al., 2014; Singh and Wurtele, 2020; Li et al., 2021). How orphan genes can impact phenotypic traits requires a mechanistic understanding of how newly emerged genes can modulate pre-existing biological processes. However, due to the nature of orphan genes, as newly emerged genes with no existing homologs, these mechanisms will be unique to each orphan gene.

Our prior characterizations have established that the overexpression of *QQS* increases protein content and decreases starch content in Arabidopsis, and in other crops (*e.g.*, rice, corn, soybean, tobacco and potato) (Li et al., 2009; Li and Wurtele, 2015; Li et al., 2015; Tanvir et al., 2022a; Tanvir et al., 2022b). Moreover, we have shown that these modifications in protein and starch content are facilitated by the interaction of the QQS protein with the trimeric transcriptional regulatory factor, Nuclear Factor Y (NF-Y) (Li et al., 2015). NF-Y is a protein complex, which mediates its regulatory function by binding to the CCAAT boxes of gene promoters (Laloum et al., 2013), and it is composed of NF-YA, NF-YB, and NF-YC subunits. We have specifically determined that QQS physically interacts with one of the paralogs of the NF-YC subunits (specifically AtNF-YC4), which mediates the regulation of C and N allocation in Arabidopsis and in multiple plant species (Li et al., 2009; Li and Wurtele, 2015; Li et al., 2015; O’Conner et al., 2018; Qi et al., 2019; O’Conner et al., 2021; Tanvir et al., 2022a; Tanvir et al., 2022b). To further refine the mechanistic model for the action of orphan genes, such as *QQS*, we developed algal-based and yeast-based, single-cell genetic systems to test the impact of the QQS protein and its interacting partners. These refactored single-cell organisms offer genetically simpler systems (*i.e.*, single copy genes encoding for NF-YA, NY-YB and NF-YC), and we monitored the effect of the QQS protein expression on the readily tractable phenotype, *i.e.*, the allocation of C and N. The results indicate that QQS and NF-YC (called HAP5 in yeast) can increase protein content and decrease polysaccharide content (*i.e.*, starch in *C. reinhardtii* or glycogen in yeast), while not significantly affecting growth. This is similar to the effect of QQS and the NF-YC4 homologs that were observed in crop plants (Li and Wurtele, 2015; Li et al., 2015; O’Conner et al., 2018; O’Conner et al., 2021; Tanvir et al., 2022a; Tanvir et al., 2022b). Moreover, these data establish that QQS is functional in a simple single-cell photosynthetic organism (*i.e.*, *C. reinhardtii*), most likely interacting with the NF-YC homolog in this model organism, and thereby modulating C/N partitioning. Therefore, these findings provide new research vehicles for dissecting the basic mechanisms of *QQS* function and expands the target species that can be used for the biotechnological application of *QQS* beyond crops (*i.e.*, soybean, rice, maize, or tobacco) (Li and Wurtele, 2015; Li et al., 2015; O’Conner et al., 2018; O’Conner et al., 2021; Tanvir et al., 2022a; Tanvir et al., 2022b).

**Figure 7:**
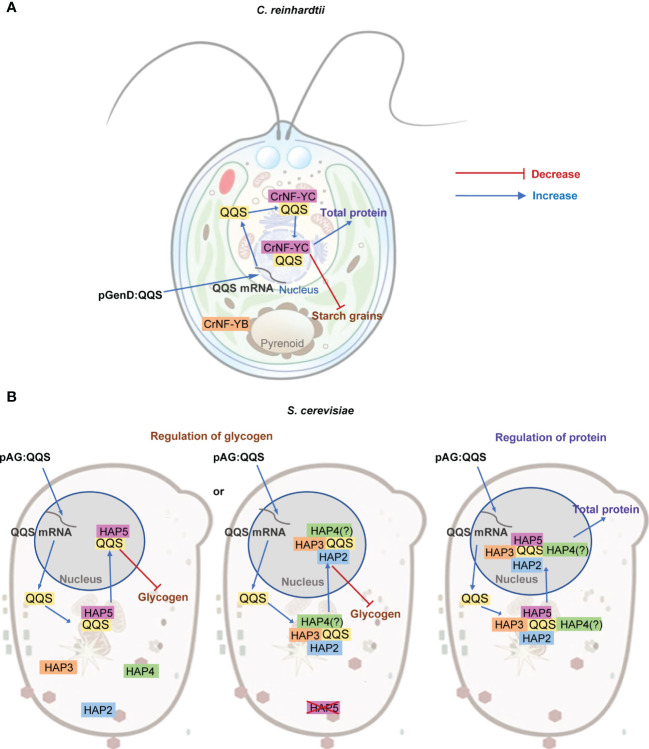
Proposed model for the interaction between QQS and NF-Y subunits in single-cell organisms. **(A)** In *C. reinhardtii*, regulation of C and N allocation appears to be *via* the interaction between QQS and the NF-YC subunit that functions in the nucleus, and does not need the NF-YA subunit. **(B)** In *S. cerevisiae*, QQS appears to regulate glycogen level *via* the interaction of QQS with either only HAP5 or with HAP2, HAP3 or HAP4, and affect protein content *via* the interaction with the HAP complex that requires HAP2, HAP3, HAP4 and HAP5.

Both the refactored single-cell organisms developed herein generate C/N phenotype alterations that we had previously observed in plant transgenic experiments (Li et al., 2009; Li and Wurtele, 2015; Li et al., 2015; O’Conner et al., 2018; Qi et al., 2019; O’Conner et al., 2021; Tanvir et al., 2022a; Tanvir et al., 2022b). However, these newly developed refactored single-cell systems provide new mechanistic insights on how *QQS* appears to affect C and N allocation. Specifically, we had previously defined that two regions of the QQS protein, located between aa 1-12 and aa 41-59, bind to a domain of AtNF-YC4, located between aa 73-162 (Li et al., 2015). We therefore hypothesized that QQS binds to AtNF-YC4, which subsequently may bind to NF-YA and/or NF-YB to alter C and N allocation (Li et al., 2015). Moreover, based on the high sequence similarity between these two regions of the QQS protein (*i.e.*, aa 5-11 and aa 41-49) and NF-YB residues between aa 51-57 and aa 62-70, we further hypothesized that QQS may mimic and thereby replace NF-YB in generating a novel NF-Y complex (*i.e.*, QQS/NF-YC/NF-YA complex) (Qi et al., 2019). However, as shown in this study, the fact that the expression of *QQS* in *C. reinhardtii* can still alter C and N allocation, despite the absence of a NF-YA subunit in this organism indicates that the QQS protein can still interact with the *C. reinhardtii* NF-YC4 homolog (*i.e.*, CrNF-YC4 encoded by Cre12.g556400) (**Figure 7A**).

The refactored yeast system offered additional capabilities to systematically dissect the effect of QQS. Specifically, whereas in *C. reinhardtii* we could evaluate the need for the NF-YA subunit in the QQS effect, in yeast, we were able to evaluate the need of every NF-Y subunit, either individually or in combination. These yeast data support parallel mechanisms for the regulation of glycogen and protein content by *QQS* expression in this organism (**Figure 7B**). Consistent with the mechanistic model gleaned from *C. reinhardtii* system, the yeast data also indicate interactions between QQS and the NF-YC subunit (*i.e.*, the HAP5 subunit of yeast). Moreover, the data obtained with the yeast mutant strains also support the conclusion that NF-YA (*i.e.*, HAP2) is not required for this interaction to regulate glycogen content. However, in the absence of the HAP5 subunit, HAP2, HAP3 or HAP4 subunits become significant in supporting the effect of QQS to regulate glycogen levels (**Figure 7B**). Therefore, we conclude that the genetic interactions that affect changes in protein content are between QQS and HAP5, with additional involvement of HAP2, HAP3 or HAP4.

The yeast strains that have been developed herein can be used as platforms to explore the role of the functionality of the large number of NF-YA, NF-YB and NF-YC Arabidopsis paralogs in affecting C and N allocation in the context of the *QQS* orphan gene. Using synthetic biological technologies that have been widely utilized to reconstitute biological processes, for example, human metabolic pathways (Carreras et al., 1997; Hopwood, 1997; Staunton and Weissman, 2001) or plant lipid metabolic pathways (Campbell et al., 2019; Stenback et al., 2022), one can readily envision strategies that systematically replace individual NF-YA, NF-YB and NF-YC Arabidopsis paralogs into the strains expressing *QQS*, and thereby individually evaluate how these different combinations of NF-Y subunits can affect the C and N allocation phenotypes. For example, such synthetic biology strategies would provide experimental evidence to support the “mimicry-model” for orphan gene function, generated from the experiments that have provided a mechanistic understanding of QQS function, which impacts a variety of different phenotypes.

Specifically, in Arabidopsis, the LEAFY COTYLEDON1 (LEC1) protein, which is the homolog of the yeast HAP3, has previously been shown to be a regulator of fatty acid biosynthesis (Kwong et al., 2003; Mu et al., 2008). Thus, whereas in yeast, QQS appears to indirectly interact with HAP3, these interactions affect changes in protein and glycogen content, and not fatty acid content. These findings indicate the molecular intricacy of evolutionarily integrating an orphan gene, such as *QQS*, into a complex metabolic system. Specifically, multiple metabolic outcomes can be affected by orphan genes, depending on the context of its integration. In the case of QQS, because its interactions mimicked the binding among NF-Y subunits, a regulatory factor that impacts many outcomes, the effect of QQS enabled the modification of multiple biological outcomes, facilitating the adaptation of Arabidopsis to new selection pressures, which thereby favoring the evolutionary maintenance of QQS.

Although this study has shown that yeast and *C. reinhardtii* are very useful hosts for reconstituting the function of QQS in regulating the C/N phenotype, there are certain limits to the utility of these hosts to generally understand the functionality of other orphan genes. For example, these two hosts express homologs of the NF-Y complex, which is the partner interactor, through which QQS mediates its effect in Arabidopsis. Furthermore, we evaluated the effect of QQS on a phenotype (*i.e.*, C/N allocation) that is shared by Arabidopsis, and yeast and *C. reinhardtii*. However, we could not utilize these hosts if we had decided to monitor the effect of QQS on phenotypes associated with enhanced defenses from insect pests or pathogens (Qi et al., 2019; Tanvir et al., 2022a). Thus, in general for a host to be useful in dissecting the functionality of an orphan gene, it has to express a homolog of the interactor that mediates the effect of the orphan gene, and the heterologous host has to express a phenotype that is affected by the orphan gene in its native host.

## Data availability statement 

The datasets presented in this study can be found in online repositories. The names of the repository/repositories and accession number(s) can be found in the article/**Supplementary Material**.

## Author contributions

LL designed the study. AT, KS, AC, WZ, YW, and DS performed research. LW, WF, AT, KS, AC, RT, JZ, SC, DW, YM, MS, BN, and LL analyzed data. LW and LL prepared the manuscript. All authors contributed to the article and approved the submitted version.
