# Supplementary material for: Single-cell genetic models to evaluate orphan gene function: The case of QQS regulating carbon and nitrogen allocation
Source: Front Plant Sci. 2023 Mar 27;14:1126139. doi: 10.3389/fpls.2023.1126139 (PMC10084940; doi:10.3389/fpls.2023.1126139)
Supplement: Supplementary file 1 [file DataSheet_1.pdf]

Supplementary Material for

Single-cell genetic models to evaluate orphan gene function: the case of QQS regulating carbon and nitrogen allocation

Lei Wang[1], Andrew J. Tonsager[2,3,4†], Wenguang Zheng[5], Yingjun Wang[5], Dan Stessman[5], Wei Fang[5], Kenna E. Stenback[2,3,4†], Alexis Campbell[2,3,4], Rezwan Tanvir[1], Jinjiang Zhang[1,6†], Samuel Cothron[1], Dongli Wan[7], Yan Meng[8], Martin H. Spalding[5], Basil J. Nikolau[2,3,4] and Ling Li[1]*

[1] Department of Biological Sciences, Mississippi State University, Mississippi State, MS, USA, [2] Roy J. Carver Department of Biochemistry, Biophysics, and Molecular Biology, Iowa State University, Ames, IA, USA, [3] Engineering Research Center for Biorenewable Chemicals, Iowa State University, Ames, IA, USA, [4] Center for Metabolic Biology, Iowa State University, Ames, IA, USA, [5] Department of Genetics, Development and Cell Biology, Iowa State University, Ames, IA, USA, [6] Mississippi School for Mathematics and Science, Columbus, MS, USA, [7] Institute of Grassland Research, Chinese Academy of Agricultural Sciences, Hohhot, China, [8] Department of Agriculture, Alcorn State University, Lorman, MS, USA

* CORRESPONDENCE

Ling Li liling@biology.msstate.edu

† Present address:

Andrew J. Tonsager, Department of Biochemistry and Molecular Biology, Colorado State University, Fort Collins, CO, USA; Kenna E. Stenback, Department of Biological Chemistry and Molecular Pharmacology, Blavatnik Institute, Harvard Medical School, Boston, MA, USA; Jinjiang Zhang, Center for Cognitive Neuroscience, Duke University, Durham, NC, USA; Samuel Cothron, Graduate School of Genome Science and Technology, University of Tennessee, Knoxville, TN, USA

Supplementary Figures

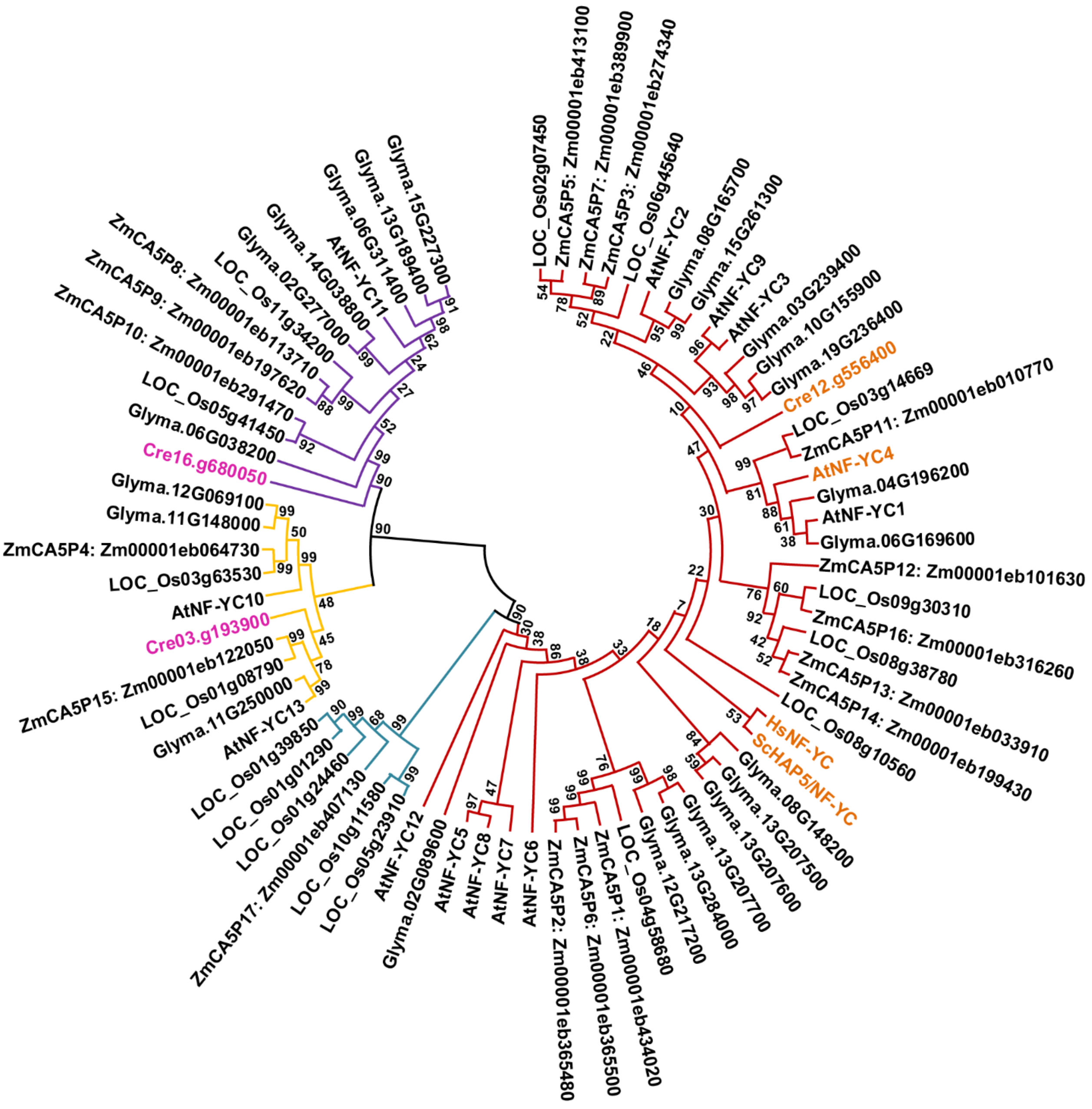

FIGURE S1 Phylogenetic analysis of NF-YC proteins, based on NF-YC region (residues 73-162 of AtNF-YC4) binding to QQS. The phylogenetic tree was constructed using the Neighbor-Joining method (1000 bootstrap replicates) with MEGA software, version X (Kumar *et al*., 2018). The percentages of replicate trees in which the associated taxa clustered together in the bootstrap

test (1,000 replicates) are shown next to the branches. Seventy-four proteins from seven species (*Saccharomyces cerevisiae* (1), *Homo sapiens* (1), *Chlamydomonas reinhardtii* (3), *Arabidopsis thaliana* (13), *Oryza sativa* (16), *Glycine max* (23), *Zea mays* (17)) were identified by sequence homology (see Supplementary Table S1). The *C. reinhardtii*, yeast, and human homologs of the Arabidopsis NF-YC4 are identified with brown-colored text. The other two NF-YC homologs in *C. reinhardtii* are identified with pink-colored text. The low bootstrap values of some of the clades are likely due to the short length of the protein sequences used to construct this tree; statistical confidence in the four major clades is supported by bootstrap values over 90%.

```
AtNF-YC4      --------------------------------------------MDNNN--NNNNQQPP-   13
CrNF-YC       --------------------------------------------------------MGD-   3
HAP5          MTDRNFSPQQGQGPQESLPEGPQPSTMIQREEMNMPRQYSEQQQLQENEGEGENTRLPVS   60
HsNF-YC       ------------------------------------------------------------   0

AtNF-YC4      --------PTSVYPPGSAVTTVIPPPPSGSASIVTGG---------------GATY----   46
CrNF-YC       --------QYNYYP-GG----------------YTGG---------------IP------   17
HAP5          EEEFRMVQELQAIQ-AGHDQANLPPSGRGSLEGEDNGNSDGADGEMDEDDEEYDVFRNVG   119
HsNF-YC       -------------------------------MSTEGG---------------FG------   8
                                                   73 AtNF-YC4 region required to bind to QQS
AtNF-YC4      HHLLQQQQQLQMFWTYQRQEIEQV---------NDFKNHQLPLARIKKIMKADEDVRMIS   98
CrNF-YC       --PNHHQAEALKSFWQAQLVEVSEVP----PDPTVFKNHQLPLARIKKIMKSDEDVRMIS   71
HAP5          QGLVGHYKEIMIRYWQELINEIESTNEPGSEHQDDFKSHSLPFARIRKVMKTDEDVKMIS   179
HsNF-YC       GTSSSDAQQSLQSFWPRVMEEIRNLT------VKDFRVQELPLARIKKIMKLDEDVKMIS   62
                          AtNF-YC4 region required to bind to QQS
AtNF-YC4      AEAPILFAKACELFILELTIRSWLHAEENKRRTLQKNDIAAAITRTDIFDFLVDIVPREE   158
CrNF-YC       AEAPVLFAKACEMFILELTLRSWMHAEENKRRTLQRNDVAAAITKTDIFDFLIDIVPRED   131
HAP5          AEAPIIFAKACEIFITELTMRAWCVAERNKRRTLQKADIAEALQKSDMFDFLIDVVPRRP   239
HsNF-YC       AEAPVLFAKAAQIFITELTLRAWIHTEDNKRRTLQRNDIAMAITKFDQFDFLIDIVPRDE   122
                 162
AtNF-YC4      IKEEEDAASALGGGGMVAPA---------A-----S----------------GVPYYYPP   188
CrNF-YC       GKPEEGGAAAPGGAAPATAPS--------PAGPGGSGNQQAASAASTAAPAAAAPRPPAP   183
HAP5          LPQ---------------------------------------------------------   242
HsNF-YC       LKPPKRQEEVRQSVTPAEPVQYYFTLAQQPTAVQVQGQQQGQQTTS--------------   168

AtNF-YC4      MGQPAVPGGMMIGRPAMD-------PSGV-------YAQPPSQA----------------   218
CrNF-YC       PGMPTAPG-MFFPPPFPM-------PPGA-------LGDPSHAAAAAA----AAAVMMRP   224
HAP5          ------------------------------------------------------------   242
HsNF-YC       STTTIQPGQIIIAQPQQGQTTPVTMQVGEGQQVQIVQAQPQGQAQQAQSGTGQTMQVMQQ   228

AtNF-YC4      -------------------WQSVWQNSAGGGDDVSYGSGG-------------SSGHG-N   245
CrNF-YC       PMGVDPNLVLQYQQQILAGQAPGWPHLPGLPPPPTSQPGAAAAAAAAAAAAAAAAAAG-A   283
HAP5          ------------------------------------------------------------   242
HsNF-YC       II-TNTGEIQQIPVQLNAGQLQ-YI---RLAQPVS---GTQVVQGQIQT-LATNAQQGQR   279

AtNF-YC4      LDSQG-------------------------------------------------------   250
CrNF-YC       AAAEGQAEAAKQE-----------------------------------------------   296
HAP5          ------------------------------------------------------------   242
HsNF-YC       NASQGKPRRCLKETLQITQTEVQQGQQQFSQFTDGQRNSVQQARVSELTGEAEPREVKAT   339

AtNF-YC4      ------------------------------------------------------------   250
CrNF-YC       ------------------------------------------------------------   296
HAP5          ------------------------------------------------------------   242
HsNF-YC       GNSTPCTSSLPTTHPPSHRAGASCVCCSQPQQSSTSPPPSDALQWVVVEVSGTPNQLETH   399

AtNF-YC4      -----------------------------------------------------------    250
CrNF-YC       -----------------------------------------------------------    296
HAP5          -----------------------------------------------------------    242
HsNF-YC       RELHAPLPGMTSLSPLHPSQQLYQIQQVTMPAGQDLAQPMFIQSANQPSDGQAPQVTGD    458
```

FIGURE S2 Multisequence alignment of Arabidopsis NF-YC4 and its homologs in *C. reinhardtii*, yeast and human. The IDs are At5g63470 (AtNF-YC4), Cre12.g556400, YOR358W (HAP5), HGNC:7806. Residues highlighted in green are identical, and residues highlighted in blue are similar. Residues 73-162 of the AtNF-YC4 sequence identify the domain that binds to QQS, “AtNF-YC4 region required to bind to QQS”.

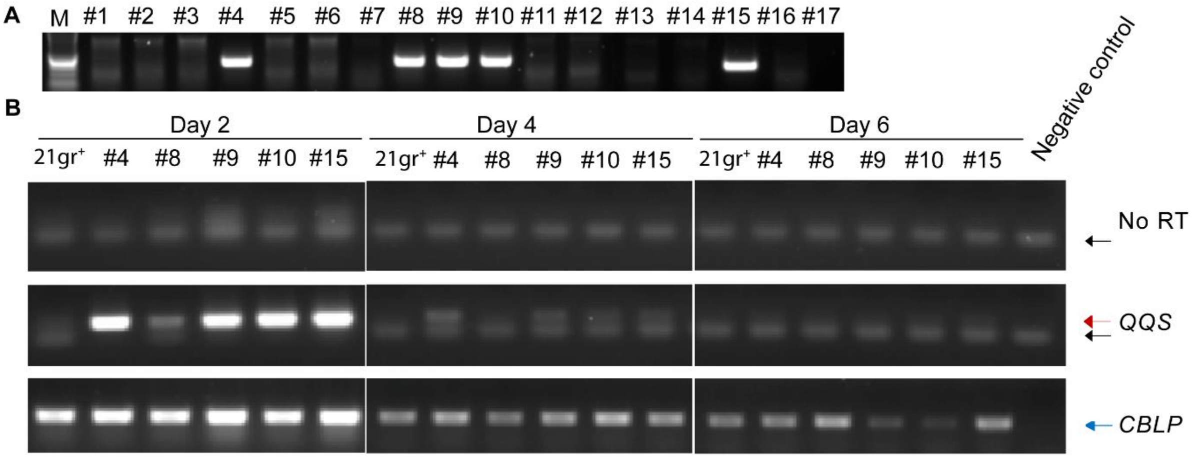

FIGURE S3 PCR-based genotyping and RT-PCR analysis of *QQS* expression in transgenic *Chlamydomonas reinhardtii* strains. (A) The presence of the *QQS* transgene was evaluated by PCR using DNA templates isolated from 17 putative *C. reinhardtii QQS-E* strains. PCR amplification was conducted with a primer situated in the promoter of the transgene and a primer situated in the *QQS* coding sequence. Strains #4, #8, #9, #10, and #15 showed the presence of the *QQS* transgene. M is the lane for DNA marker. **(B)** The expression of the *QQS* RNA assay by RT-PCR analysis using RNA isolated from the indicated strains sampled at 2, 4, and 6 days after subculturing. Strains #4, #9, #10 and #15 showed high levels of *QQS* expression on Day 2, but this level decreased on Day 4 and was undetectable above background on Day 6; *QQS* expression in strain #8 was lower than the other strains. The expression of the RNA coding for the guanine nucleotide-binding protein beta subunit-like protein (*CBLP*) was used as a control, and its expression pattern was similar to the expression pattern of the *QQS* RNA. Control assays used RNAs isolated from non-transgenic 21gr^{+} strain. The black, red, blue arrows indicate the bands accounting for primer dimers, QQS gene, and CBLP control respectively. The primers used in these analyses are listed in Supplementary Table S1.

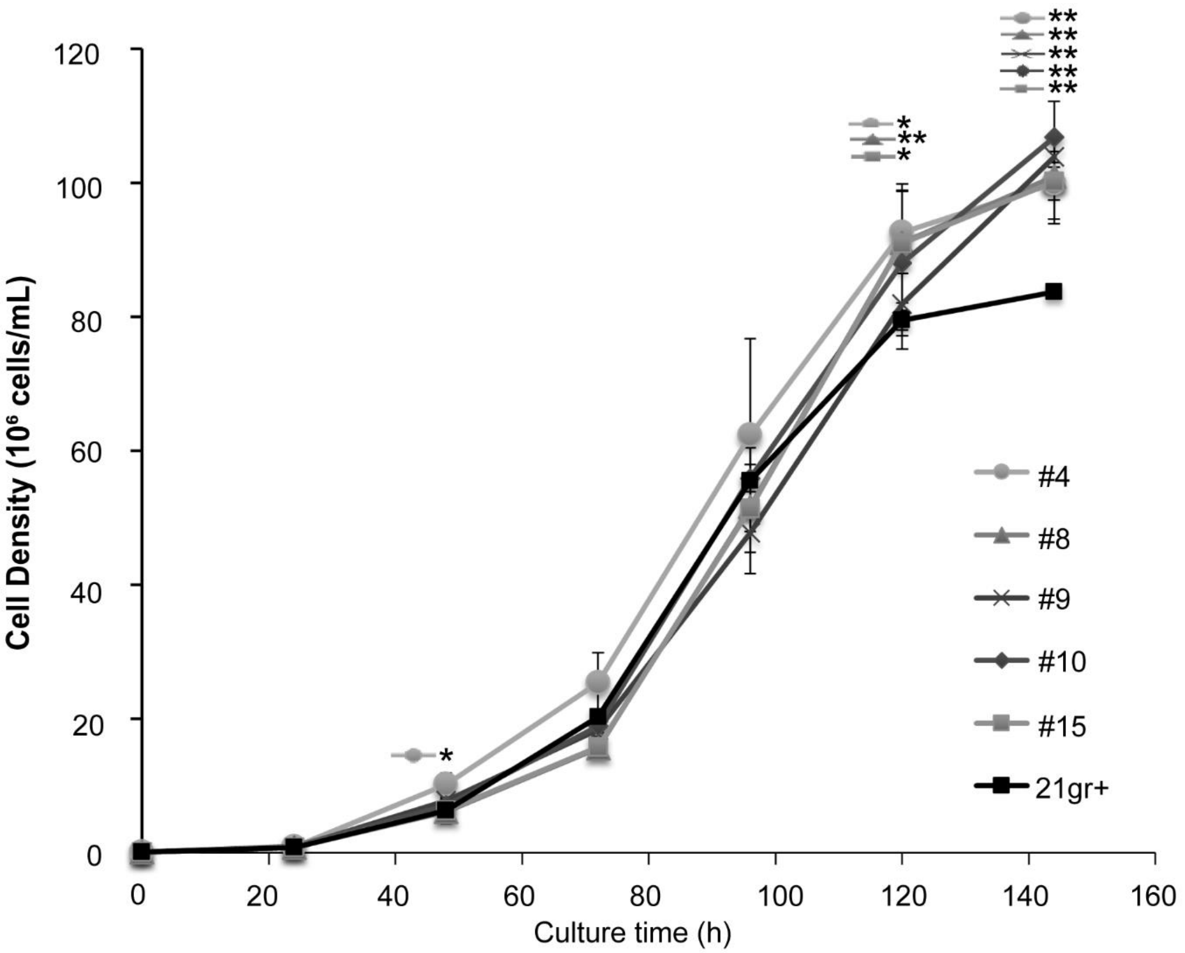

FIGURE S4 Growth of *Chlamydomonas reinhardtii* cultures expressing *QQS*. The growth of the indicated strains was monitored by measuring cell density with a Z1 Coulter Particle Counter (Beckman Coulter Inc, USA). All data points are the mean ± SE (standard error), $n = 3$. Statistical significance relative to the control strain was calculated with Student's *t*-test and is indicated: $**P < 0.01$, $*P < 0.05$.

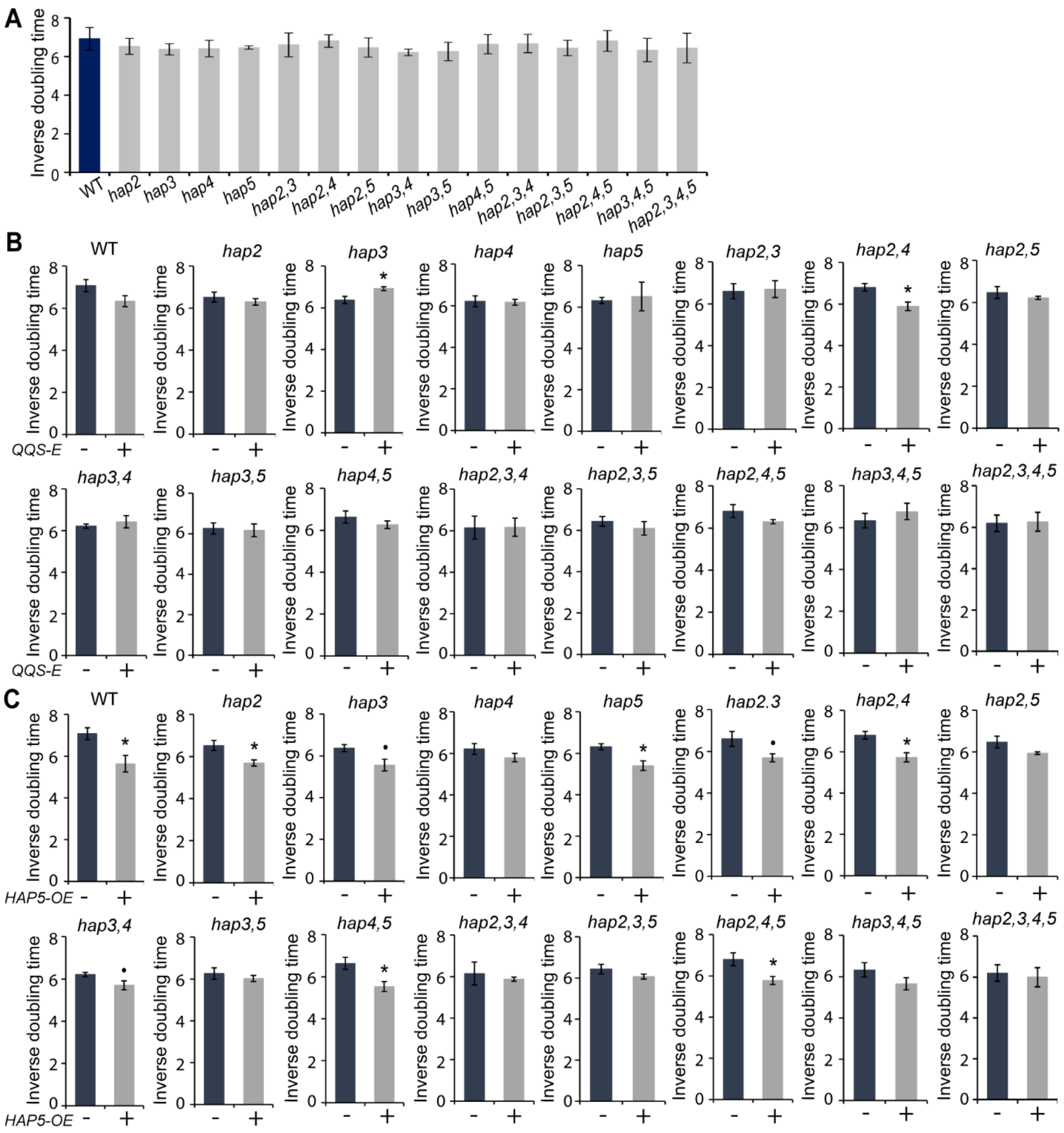

FIGURE S5 Growth of yeast *hap* cultures in the absence and presence of transgenic *QQS-E* or *HAP5-OE*. (A) The growth of each mutant strain is compared to the growth of the control WT strain. **(B)** The growth of each mutant strain that is expressing the *QQS* transgene (*QQS-E*) is compared to the growth of the mutant strain. **(C)** The growth of each mutant strain that is

overexpressing the *HAP5* gene (*HAP5-OE*) is compared to the growth of the mutant strain. Yeast haploid strains carrying *hap2*, *hap3*, *hap4*, or *hap5* single mutants, or all possible double, triple and quadruple mutant combinations were generated as described in Figure 3 and were used in these experiments. Growth of all strains was determined as the inverse of doubling time, and each graph plots the mean ± SE, $n = 3$. Statistical significance relative to the control strain was calculated with Student's *t*-test and is indicated: *$P < 0.05$, •$P < 0.1$.

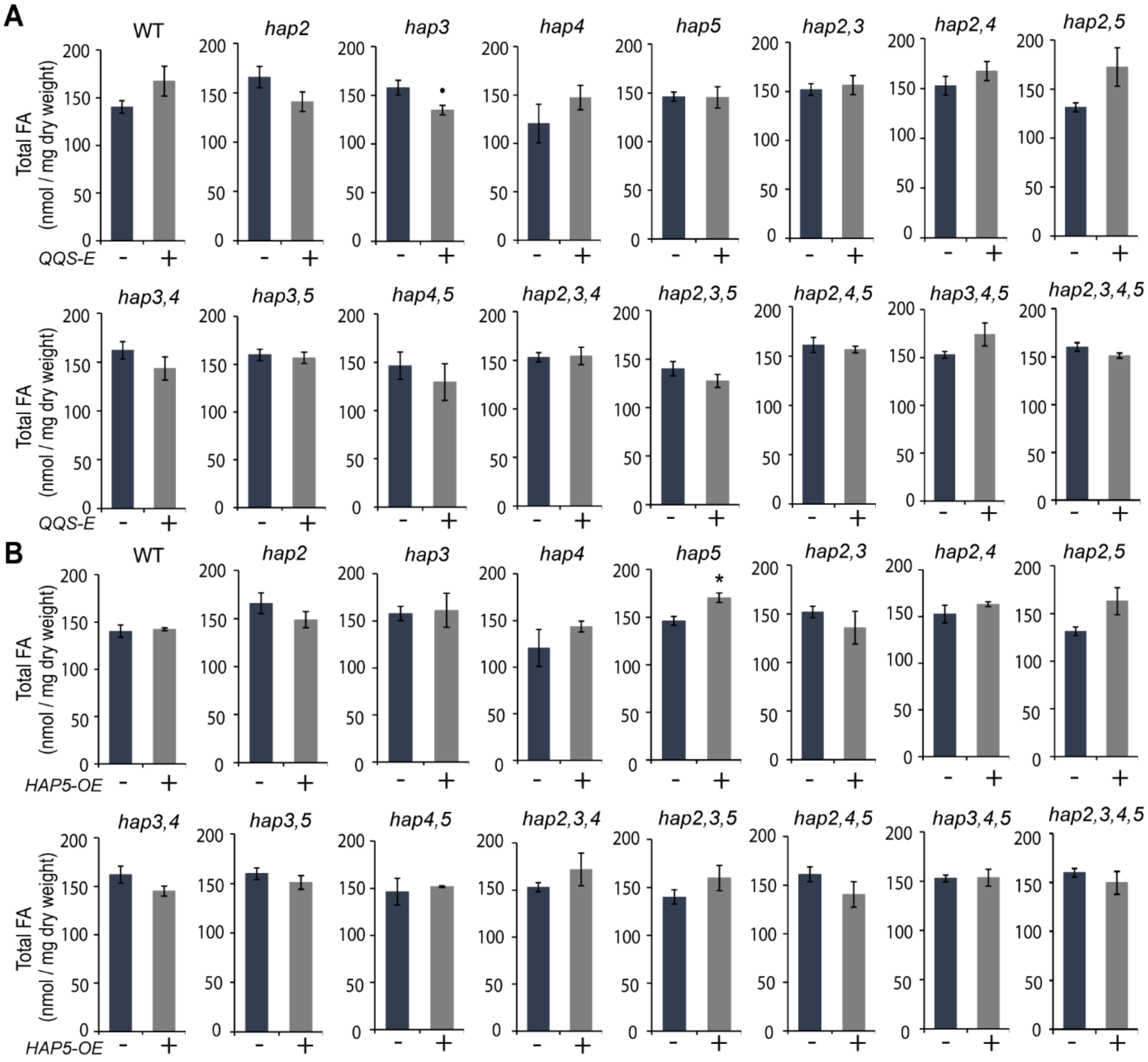

FIGURE S6 The effect of transgenic *QQS-E* and *HAP5-OE* on fatty acid content of yeast *hap* strains. (A) *QQS-E*. **(B)** *HAP5-OE*. Yeast haploid strains carrying *hap2*, *hap3*, *hap4,* or *hap5* single mutants, or all possible double, triple and quadruple mutant combinations were generated as described in Figure 3 and were used in these experiments. The fatty acid content of each strain was determined as described in the Materials and Methods, and each graph plots the mean ± SE,

$n = 3$. Statistical significance relative to the control strain was calculated with Student's *t*-test and is indicated: *$P < 0.05$, •$P < 0.1$.

Supplementary Tables

TABLE S1 Primers used in the experiments.

Primer name	Primer (5'-3')	Purpose
Cr-RT-QQS-F	ATGAAGACCAATAGAGAGCA	Check *QQS* expression in *C. reinhardtii* by RT-PCR
Cr-RT-QQS-R	TCAGTAGTTGTAGAACTGAA	Check *QQS* expression in *C. reinhardtii* by RT-PCR
Cr-PCR-F	TCGTCAGAAACACGTCTCC	PCR amplification with a primer from promoter and a specific *QQS* primer
Cr-PCR-R	CCGACCCATGATATGACCCT	PCR amplification with a primer from promoter and a specific *QQS* primer
Cr-RT-CBLP-F	ATGTGCTGTCCGTGGCTTTC	Internal control gene in *C. reinhardtii* for RT-PCR
Cr-RT-CBLP-R	CAGACCTTGACCATCTTGTCCC	Internal control gene in *C. reinhardtii* for RT-PCR
HAP5gateway-F	GGGGACAAGTTTGTACAAAAAAGCAGGC TTCATGACTGATAGGAATTTCTCACCA	Cloning for overexpression
HAP5gateway-R	GGGGACCACTTTGTACAAGAAAGCTGGG TCTCATTGTGGAAGAGGTCTTCTAGG	Cloning for overexpression
KanB-R	CTGCAGCGAGGAGCCGTAAT	Genotyping for mutants
KanC-F	TGATTTTGATGACGAGCGTAAT	Genotyping for mutants
hap2-A-F	ACTGAATGCAAACTACTCTTGAACC	Paired with KanB-R, Genotyping
hap2-D-R	CATTTTTAAATACCATTTTGCCTCA	Paired with KanC-F, Genotyping
hap3-A-F	TGTGGCCCTTTACTGCTACTAATAC	Paired with KanB-R, Genotyping
hap3-D-R	TAGGTGTCCTAGTAACCGTATCAGC	Paired with KanC-F, Genotyping
hap4-A-F	TTAATTCCTTCACCTCTCTAAACCC	Paired with KanB-R, Genotyping
hap4-D-R	AACGGATATGTGAAAATGCTCTTAG	Paired with KanC-F, Genotyping
hap5-A-F	ACTTAGTACGTGATAGTGCCCAAAG	Paired with KanB-R, Genotyping
hap5-D-R	TTCATGATTTCTTTGCTGACAATTA	Paired with KanC-F, Genotyping
QQS-E-F	ATGAAGACCAATAGAGAGCA	Cloning and genotyping in yeast
QQS-E-R	TCAGTAGTTGTAGAACTGAA	Cloning and genotyping in yeast
HAP5-OE-F	ATGACTGATAGGAATTTCTCACCA	Genotyping
HAP5-OE-R	TCATTGTGGAAGAGGTCTTCTAGG	Genotyping

TABLE S2 Identification of *Chlamydomonas reinhardtii* NF-YA, NF-YB, and NF-YC homologs using Arabidopsis query sequences.

Query Locus ID	Gene	Family	Length (aa)	*C. reinhardtii* Homolog 1	Homolog 2	Homolog 3	Matched sequence *A.t.* (aa)	*C.r.* (aa)
AT5G12840	NF-YA1	NF-YA	272	No match				
AT3G05690	NF-YA2	NF-YA	295	No match				
AT1G72830	NF-YA3	NF-YA	341	No match				
AT2G34720	NF-YA4	NF-YA	198	No match				
AT1G54160	NF-YA5	NF-YA	308	No match				
AT3G14020	NF-YA6	NF-YA	308	No match				
AT1G30500	NF-YA7	NF-YA	190	No match				
AT1G17590	NF-YA8	NF-YA	328	No match				
AT3G20910	NF-YA9	NF-YA	303	No match				
AT5G06510	NF-YA10	NF-YA	269	No match				
AT2G38880	NF-YB1	NF-YB	164	Cre02.g079200	Cre07.g341800	Cre17.g739450	10-110	6-106
AT5G47640	NF-YB2	NF-YB	190	Cre02.g079200	Cre07.g341800	Cre17.g739450	26-118	16-108
AT4G14540	NF-YB3	NF-YB	161	Cre02.g079200	Cre07.g341800	Cre17.g739450	8-120	2-116
AT1G09030	NF-YB4	NF-YB	139	Cre02.g079200	Cre17.g739450	Cre07.g341800	3-108	17-120
AT2G47810	NF-YB5	NF-YB	190	Cre02.g079200	Cre07.g341800	Cre17.g739450	49-152	15-118
AT5G47670	NF-YB6	NF-YB	234	Cre02.g079200	Cre07.g341800	Cre17.g739450	48-160	7-119
AT2G13570	NF-YB7	NF-YB	215	Cre02.g079200	Cre07.g341800	Cre17.g739450	35-134	16-115
AT2G37060	NF-YB8	NF-YB	173	Cre02.g079200	Cre07.g341800	Cre17.g739450	27-131	14-118
AT1G21970	NF-YB9	NF-YB	238	Cre02.g079200	Cre07.g341800	Cre17.g739450	57-151	15-109
AT3G53340	NF-YB10	NF-YB	176	Cre02.g079200	Cre07.g341800	Cre17.g739450	26-123	14-111
AT2G27470	NF-YB11	NF-YB	275	Cre07.g341800	Cre02.g079200	Cre12.g556400	10-98	13-103
AT5G08190	NF-YB12	NF-YB	163	Cre17.g739450	Cre02.g079200	Cre07.g341800	24-84	1-61
AT5G23090	NF-YB13	NF-YB	159	Cre17.g739450	Cre02.g079200	Cre07.g341800	24-102	1-79
AT3G48590	NF-YC1	NF-YC	234	Cre12.g556400	Cre16.g680050		47-147	29-133
AT1G56170	NF-YC2	NF-YC	199	Cre12.g556400	Cre16.g680050		70-158	45-133
AT1G54830	NF-YC3	NF-YC	217	Cre12.g556400	Cre16.g680050		51-150	28-131
AT5G63470	NF-YC4	NF-YC	250	Cre12.g556400	Cre16.g680050		40-160	13-133
AT5G50490	NF-YC5	NF-YC	186	Cre12.g556400	Cre16.g680050		7-118	19-133

AT5G50480	NF-YC6	NF-YC	202	Cre12.g556400	Cre16.g680050		29-135	9-132
AT5G50470	NF-YC7	NF-YC	212	Cre12.g556400	Cre16.g680050		46-148	27-130
AT5G27910	NF-YC8	NF-YC	187	Cre12.g556400	Cre16.g680050		10-110	16-125
AT1G08970	NF-YC9	NF-YC	231	Cre12.g556400	Cre16.g680050		63-160	30-131
AT1G07980	NF-YC10	NF-YC	206	Cre12.g556400	Cre16.g680050		109-186	51-128
AT3G12480	NF-YC11	NF-YC	293	Cre16.g680050	Cre12.g556400		11-90	10-89
AT5G38140	NF-YC12	NF-YC	195	Cre12.g556400			47-144	25-128
AT5G43250	NF-YC13	NF-YC	130	Cre12.g556400	Cre16.g680050	Cre03.g193900	11-89	51-128

TABLE S3 Expression level of *CrNF-Y* genes in *Chlamydomonas reinhardtii*.

Locus ID	Gene expression level[1]	Matched family	Protein length (aa)
Cre02.g079200	~14-28 RPKM (very low)	AtNF-YB1-10	209
Cre07.g341800	~ 1.5-2.8 RPKM (very low)	AtNF-YB11	278
Cre17.g739450	~ 7.8-10.5 RPKM (very low)	AtNF-YB12-13	189
Cre12.g556400	~ 6.3-8.5 RPKM (very low)	AtNF-YC1-10,12-13	296
Cre16.g680050	~ 10-20 RPKM (very low)	AtNF-YC11	649
Cre03.g193900	~ 2.5-8 RPKM (very low)	AtNF-YC13	231

[1]Information from Augustus 5.0 (http://augustus.gobics.de/predictions/chlamydomonas/) as described in (Fang et al., 2012). Rubisco (RBCS) gene transcript level is about 2500 to 3000 RPKM (Reads Per Kilobase of transcript, per Million mapped reads (Mortazavi et al., 2008)), and any gene with a transcript level lower than 100 RPKM is considered to have a very low expression level which is hard to detect by northern blot method.

TABLE S4 NF-YC homologs from *Saccharomyces cerevisiae*, *Homo sapiens*, *Chlamydomonas reinhardtii*, *Arabidopsis thaliana*, *Oryza sativa*, *Glycine max*, and *Zea mays* that were used for phylogenetic analysis in Supplementary Figure S1.

**Species**	**Gene name**	**Locus**	**Data source**
Homo sapiens	HUMAN NF-YC	HGNC: 7806	HGNC
S. cerevisiae	HAP5	HAP5 / YOR358W	SGD
C. reinhardtii	Cre03.g193900	Cre03.g193900	PlantTFDB
C. reinhardtii	Cre12.g556400	Cre12.g556400	PlantTFDB
C. reinhardtii	Cre16.g680050	Cre16.g680050	PlantTFDB
Arabidopsis thaliana	AtNF-YC1	At3g48590	PlantTFDB
Arabidopsis thaliana	AtNF-YC2	At1g56170	PlantTFDB
Arabidopsis thaliana	AtNF-YC3	At1g54830	PlantTFDB
Arabidopsis thaliana	AtNF-YC4	At5g63470	PlantTFDB
Arabidopsis thaliana	AtNF-YC5	At5g50490	PlantTFDB
Arabidopsis thaliana	AtNF-YC6	At5g50480	PlantTFDB
Arabidopsis thaliana	AtNF-YC7	At5g50470	PlantTFDB
Arabidopsis thaliana	AtNF-YC8	At5g27910	PlantTFDB
Arabidopsis thaliana	AtNF-YC9	At1g08970	PlantTFDB
Arabidopsis thaliana	AtNF-YC10	At1g07980	PlantTFDB
Arabidopsis thaliana	AtNF-YC11	At3g12480	PlantTFDB
Arabidopsis thaliana	AtNF-YC12	At5g38140	PlantTFDB
Arabidopsis thaliana	AtNF-YC13	At5g43250	PlantTFDB
Oryza sativa	LOC_Os01g01290	LOC_Os01g01290	PlantTFDB
Oryza sativa	LOC_Os01g08790	LOC_Os01g08790	PlantTFDB
Oryza sativa	LOC_Os01g24460	LOC_Os01g24460	PlantTFDB
Oryza sativa	LOC_Os01g39850	LOC_Os01g39850	PlantTFDB
Oryza sativa	LOC_Os02g07450	LOC_Os02g07450	PlantTFDB
Oryza sativa	LOC_Os03g14669	LOC_Os03g14669	PlantTFDB
Oryza sativa	LOC_Os03g63530	LOC_Os03g63530	PlantTFDB
Oryza sativa	LOC_Os04g58680	LOC_Os04g58680	PlantTFDB
Oryza sativa	LOC_Os05g23910	LOC_Os05g23910	PlantTFDB
Oryza sativa	LOC_Os05g41450	LOC_Os05g41450	PlantTFDB
Oryza sativa	LOC_Os06g45640	LOC_Os06g45640	PlantTFDB
Oryza sativa	LOC_Os08g10560	LOC_Os08g10560	PlantTFDB
Oryza sativa	LOC_Os08g38780	LOC_Os08g38780	PlantTFDB
Oryza sativa	LOC_Os09g30310	LOC_Os09g30310	PlantTFDB

Oryza sativa	LOC_Os10g11580	LOC_Os10g11580	PlantTFDB
Oryza sativa	LOC_Os11g34200	LOC_Os11g34200	PlantTFDB
Glycine max	Glyma.02G089600	Glyma.02G089600	PlantTFDB
Glycine max	Glyma.02G277000	Glyma.02G277000	PlantTFDB
Glycine max	Glyma.03G239400	Glyma.03G239400	PlantTFDB
Glycine max	Glyma.04G196200	Glyma.04G196200	PlantTFDB
Glycine max	Glyma.06G038200	Glyma.06G038200	PlantTFDB
Glycine max	Glyma.06G169600	Glyma.06G169600	PlantTFDB
Glycine max	Glyma.06G311400	Glyma.06G311400	PlantTFDB
Glycine max	Glyma.08G148200	Glyma.08G148200	PlantTFDB
Glycine max	Glyma.08G165700	Glyma.08G165700	PlantTFDB
Glycine max	Glyma.10G155900	Glyma.10G155900	PlantTFDB
Glycine max	Glyma.11G148000	Glyma.11G148000	PlantTFDB
Glycine max	Glyma.11G250000	Glyma.11G250000	PlantTFDB
Glycine max	Glyma.12G069100	Glyma.12G069100	PlantTFDB
Glycine max	Glyma.12G217200	Glyma.12G217200	PlantTFDB
Glycine max	Glyma.13G189400	Glyma.13G189400	PlantTFDB
Glycine max	Glyma.13G207500	Glyma.13G207500	PlantTFDB
Glycine max	Glyma.13G207600	Glyma.13G207600	PlantTFDB
Glycine max	Glyma.13G207700	Glyma.13G207700	PlantTFDB
Glycine max	Glyma.13G284000	Glyma.13G284000	PlantTFDB
Glycine max	Glyma.14G038800	Glyma.14G038800	PlantTFDB
Glycine max	Glyma.15G227300	Glyma.15G227300	PlantTFDB
Glycine max	Glyma.15G261300	Glyma.15G261300	PlantTFDB
Glycine max	Glyma.19G236400	Glyma.19G236400	PlantTFDB
Zea mays	ZmCA5P1: Zm00001eb434020	GRMZM2G074773 (Version 2)	PlantTFDB
		Zm00001eb434020 (Version 5)	maizeGDB
Zea mays	ZmCA5P2: Zm00001eb365480	GRMZM2G479610 (Version 2)	PlantTFDB
		Zm00001eb365480 (Version 5)	maizeGDB
Zea mays	ZmCA5P3: Zm00001eb274340	GRMZM2G161680 (Version 2)	PlantTFDB
		Zm00001eb274340 (Version 5)	maizeGDB
Zea mays	ZmCA5P4: Zm00001eb064730	GRMZM2G083964 (Version 2)	PlantTFDB
		Zm00001eb064730 (Version 5)	maizeGDB
Zea mays	ZmCA5P5: Zm00001eb413100	GRMZM2G311316 (Version 2)	PlantTFDB
		Zm00001eb413100 (Version 5)	maizeGDB
Zea mays	ZmCA5P6: Zm00001eb365500	GRMZM2G375448 (Version 2)	PlantTFDB
		Zm00001eb365500 (Version 5)	maizeGDB

Zea mays	ZmCA5P7: Zm00001eb389900	GRMZM2G124421 (Version 2)	PlantTFDB
		Zm00001eb389900 (Version 5)	maizeGDB
Zea mays	ZmCA5P8: Zm00001eb113710	GRMZM2G105317 (Version 2)	PlantTFDB
		Zm00001eb113710 (Version 5)	maizeGDB
Zea mays	ZmCA5P9: Zm00001eb197620	GRMZM2G091433 (Version 2)	PlantTFDB
		Zm00001eb197620 (Version 5)	maizeGDB
Zea mays	ZmCA5P10: Zm00001eb291470	GRMZM2G440949 (Version 2)	PlantTFDB
		Zm00001eb291470 (Version 5)	maizeGDB
Zea mays	ZmCA5P11: Zm00001eb010770	GRMZM2G089812 (Version 2)	PlantTFDB
		Zm00001eb010770 (Version 5)	maizeGDB
Zea mays	ZmCA5P12: Zm00001eb101630	GRMZM2G022162 (Version 2)	PlantTFDB
		Zm00001eb101630 (Version 5)	maizeGDB
Zea mays	ZmCA5P13: Zm00001eb033910	GRMZM2G113127 (Version 2)	PlantTFDB
		Zm00001eb033910 (Version 5)	maizeGDB
Zea mays	ZmCA5P14: Zm00001eb199430	GRMZM2G174776 (Version 2)	PlantTFDB
		Zm00001eb199430 (Version 5)	maizeGDB
Zea mays	ZmCA5P15: Zm00001eb122050	GRMZM2G099461 (Version 2)	PlantTFDB
		Zm00001eb122050 (Version 5)	maizeGDB
Zea mays	ZmCA5P16: Zm00001eb316260	GRMZM2G078691 (Version 2)	PlantTFDB
		Zm00001eb316260 (Version 5)	maizeGDB
Zea mays	ZmCA5P17: Zm00001eb407130	GRMZM2G052499 (Version 2)	PlantTFDB
		Zm00001eb407130 (Version 5)	maizeGDB

Data source: HGNC, HUGO Gene Nomenclature Committee, https://www.genenames.org. SGD, the Saccharomyces Genome Database, https://www.yeastgenome.org. PlantTFDB, Plant Transcription Factor Database, http://planttfdb.gao-lab.org. maizeGDB, maize Genetics and Genomics Database, https://www.maizegdb.org. Zea mays Version 2: 5b (B73 RefGen_v2), Zea mays Version 5: Zm00001eb.1 (Zm-B73-REFERENCE-NAM-5.0).

References

Fang, W., Si, Y., Douglass, S., Casero, D., Merchant, S. S., Pellegrini, M., et al. (2012). Transcriptome-wide changes in *Chlamydomonas reinhardtii* gene expression regulated by carbon dioxide and the CO_2-concentrating mechanism regulator CIA5/CCM1. *Plant Cell* 24, 1876–1893. Doi: 10.1105/tpc.112.097949

Kumar, S., Stecher, G., Li, M., Knyaz, C., and Tamura, K. (2018). MEGA X: molecular evolutionary genetics analysis across computing platforms. *Mol. Biol. Evol.* 35, 1547–1549. doi: 10.1093/molbev/msy096

Mortazavi, A., Williams, B. A., McCue, K., Schaeffer, L., and Wold, B. (2008). Mapping and quantifying mammalian transcriptomes by RNA-Seq. *Nat. Methods* 5, 621-628. doi: 10.1038/nmeth.1226
